# The Mobile Element Locator Tool (MELT): population-scale mobile element discovery and biology

**DOI:** 10.1101/gr.218032.116

**Published:** 2017-11

**Authors:** Eugene J. Gardner, Vincent K. Lam, Daniel N. Harris, Nelson T. Chuang, Emma C. Scott, W. Stephen Pittard, Ryan E. Mills, The 1000 Genomes Project Consortium, Scott E. Devine

**Affiliations:** 1 Program in Molecular Medicine, University of Maryland School of Medicine, Baltimore, Maryland 21201, USA;; 2 Institute for Genome Sciences, University of Maryland School of Medicine, Baltimore, Maryland 21201, USA;; 3 Greenebaum Cancer Center, University of Maryland School of Medicine, Baltimore, Maryland 21201, USA;; 4 Department of Medicine, University of Maryland School of Medicine, Baltimore, Maryland 21201, USA;; 5 Division of Gastroenterology, Department of Medicine, University of Maryland School of Medicine, Baltimore, Maryland 21201, USA;; 6 Department of Biostatistics and Bioinformatics, Rollins School of Public Health, Emory University, Atlanta, Georgia 30322, USA;; 7 Department of Computational Medicine and Bioinformatics, University of Michigan Medical School, Ann Arbor, Michigan 48109, USA;; 8 Department of Human Genetics, University of Michigan Medical School, Ann Arbor, Michigan 48109, USA

## Abstract

Mobile element insertions (MEIs) represent ∼25% of all structural variants in human genomes. Moreover, when they disrupt genes, MEIs can influence human traits and diseases. Therefore, MEIs should be fully discovered along with other forms of genetic variation in whole genome sequencing (WGS) projects involving population genetics, human diseases, and clinical genomics. Here, we describe the Mobile Element Locator Tool (MELT), which was developed as part of the 1000 Genomes Project to perform MEI discovery on a population scale. Using both Illumina WGS data and simulations, we demonstrate that MELT outperforms existing MEI discovery tools in terms of speed, scalability, specificity, and sensitivity, while also detecting a broader spectrum of MEI-associated features. Several run modes were developed to perform MEI discovery on local and cloud systems. In addition to using MELT to discover MEIs in modern humans as part of the 1000 Genomes Project, we also used it to discover MEIs in chimpanzees and ancient (Neanderthal and Denisovan) hominids. We detected diverse patterns of MEI stratification across these populations that likely were caused by (1) diverse rates of MEI production from source elements, (2) diverse patterns of MEI inheritance, and (3) the introgression of ancient MEIs into modern human genomes. Overall, our study provides the most comprehensive map of MEIs to date spanning chimpanzees, ancient hominids, and modern humans and reveals new aspects of MEI biology in these lineages. We also demonstrate that MELT is a robust platform for MEI discovery and analysis in a variety of experimental settings.

Population-scale, whole genome sequencing (WGS) projects have rapidly expanded over the past several years (The 1000 Genomes Project Consortium 2010, 2012, 2015; Genome of the Netherlands Consortium 2014; Gudbjartsson et al. 2015; The UK10K Consortium 2015; Telenti et al. 2016). As we look to the future, projects are planned or underway to sequence many thousands of additional human genomes for studies involving population genetics, human diseases, and clinical genomics. Although new technologies such as the Illumina HiSeq X platform can produce the massive amounts of WGS data that are required for such studies, many of the analysis tools that were developed over the past decade cannot be scaled up to meet the informatics demands of these data-intensive projects. Tools that detect mobile element insertions (MEIs) are no exception, and as a consequence, MEIs are not being routinely detected in most population-scale WGS projects (e.g., Gudbjartsson et al. 2015; The UK10K Consortium 2015; Telenti et al. 2016). Thus, there is a clear need for innovative MEI discovery approaches that can address this important gap in variant detection.

MEIs should be fully discovered along with other forms of genetic variation because they can alter human traits or cause diseases when they disrupt genes. For example, at least five reported cases of hemophilia A have been linked to germline MEIs that disrupted the coagulation factor VIII (*F8*) gene (Kazazian et al. 1988; Van de Water et al. 1998; Sukarova et al. 2001; Ganguly et al. 2003), and another six cases of hemophilia B have been linked to germline MEIs that disrupted the coagulation factor IX (*F9*) gene (Vidaud et al. 1993; Wulff et al. 2000; Li et al. 2001; Mukherjee et al. 2004; Nakamura et al. 2015). Ten out of 11 (90.1%) of these insertions disrupted coding exons, while the remaining insertion caused exon “skipping” (Ganguly et al. 2003). Similar germline MEIs have been implicated in a range of other human diseases, including neurofibromatosis (Wallace et al. 1991), Duchenne muscular dystrophy (Narita et al. 1993), cystic fibrosis (Chen et al. 2008), retinitis pigmentosis (Schwahn et al. 1998), beta-thalassemia (Divoky et al. 1996; Kimberland et al. 1999; Lanikova et al. 2013), various cancers (Miki et al. 1996; Teugels et al. 2005), and other diseases (e.g., Janicic et al. 1995; Claverie-Martin et al. 2003; Watanabe et al. 2005). As above, most of these diseases were caused by MEIs that disrupted the coding exons of genes or caused exon skipping, although disease-causing MEIs also have been identified in the promoters (Lanikova et al. 2013) and untranslated regions (UTRs) of protein-coding genes (Watanabe et al. 2005). Thus, MEIs can influence human traits and diseases by disrupting a range of gene features.

MEIs also are mobilized in somatic human tissues, including epithelial cancers, suggesting that somatic MEIs might help to drive tumorigenesis (Miki et al. 1992; Iskow et al. 2010; Lee et al. 2012; Solyom et al. 2012; Shukla et al. 2013; Helman et al. 2014; Tubio et al. 2014; Doucet-O'Hare et al. 2015; Ewing et al. 2015; Rodic et al. 2015; Scott et al. 2016). Likewise, somatic MEIs are produced in at least some normal somatic tissues such as the colon and brain (Muotri et al. 2005; Coufal et al. 2009; Baillie et al. 2011; Evrony et al. 2012; Upton et al. 2015; Scott et al. 2016), where they have been linked to colorectal cancer (Scott et al. 2016), schizophrenia (Bundo et al. 2014), and Aicardi-Goutieres syndrome (Upton et al. 2015). Thus, MEIs ideally would be discovered routinely in somatic WGS projects involving normal/tumor pairs and individual cells in order to gain a better understanding of cancers and other diseases.

Three major classes of mobile elements, i.e., *Alu*, L1, and SVA elements, remain actively mobile in human genomes and continue to generate new offspring MEIs (Mills et al. 2007; Beck et al. 2010; Ewing and Kazazian 2010; Huang et al. 2010; Iskow et al. 2010; Stewart et al. 2011; Sudmant et al. 2015; The 1000 Genomes Project Consortium 2015). All three of these element classes are mobilized by the L1 retrotransposition machinery, and as a consequence, all of these elements have at least some characteristic features of L1 elements. For example, the target site duplications (TSDs) that flank new *Alu*, L1, and SVA insertions are all very similar because they are created by the same L1-encoded proteins and target-primed-reverse-transcriptase (TPRT) mechanism (Luan et al. 1993; Moran et al. 1996; Dewannieux et al. 2003; Hancks et al. 2011; Raiz et al. 2012). Likewise, interior mutations frequently are introduced into *Alu*, L1, and SVA copies by the error-prone L1 reverse transcriptase that replicates all three of these element classes (Gilbert et al. 2005). Interior mutations have been useful for identifying and tracking active subfamilies of *Alu*, L1, and SVA elements (Boissinot et al. 2000; Batzer and Deininger 2002; Wang et al. 2005; Konkel et al. 2015) and for tracking relationships between source elements and their offspring (Scott et al. 2016). Several additional hallmark features of human MEIs include (1) 3′ transductions that are caused by alternative, downstream poly(A) signals (Moran et al. 1999), (2) 5′ inversions that are caused by twin priming (Ostertag and Kazazian 2001), and (3) 5′ truncations that are caused by incomplete replication (Beck et al. 2011). Ideally, MEI discovery tools would fully detect all of these associated genetic features because such features are useful for studying the biological impact of MEIs in humans.

## Results

### Overview of MELT

As outlined above, there is an unmet need for a robust MEI discovery package that can comprehensively detect MEIs and their associated genetic features on a population scale in humans. As members of the 1000 Genomes Project, we developed the Mobile Element Locator Tool (MELT) to address this need. The 1000 Genomes Project was ideal for this purpose because we were faced with the challenge of performing MEI discovery in 2534 human genomes. This included 2504 low-coverage Illumina whole genome sequences (averaging 7.4× coverage), and 30 high-coverage Illumina whole genome sequences (averaging 60× coverage). We also sought to leverage the data that were collected across populations to construct comprehensive MEI models at each MEI site, which allowed us to more accurately discover MEI-associated features and genotypes. Finally, we wished to develop an expanded toolkit to track and study the MEIs that were discovered in these genomes. The MELT package includes a robust MEI discovery algorithm and a suite of MEI analysis tools that collectively achieve these goals.

MELT detects *Alu*, L1, and SVA MEIs by searching for signatures of discordant read pairs (DRPs) and split reads (SRs) in Illumina WGS data that are enriched at sites containing new, nonreference (non-REF) MEIs (Supplemental Fig. S1). MELT was designed to work with BAM files that are generated with the Burroughs-Wheeler Alignment tool (BWA-ALN or -MEM) (Li and Durbin 2009, 2010), since most Illumina WGS data sets are directly available in this format. MELT first scans BAM files to identify a specific type of DRP (i.e., DRPs where one mate maps to the reference genome and the other maps to an *Alu*, L1, or SVA reference mobile element sequence), thus indicating the presence of a candidate non-REF MEI at the site. MELT uses SRs to further refine the precise breakpoints and TSDs at each candidate MEI site. When applied on a population scale, MELT constructs MEI models using all of the available DRP and SR data from multiple samples to accurately discover each MEI site and its features. MELT identifies a comprehensive set of MEI-associated features, including: the chromosomal insertion site, MEI orientation, TSD, internal mutation profile, subfamily, and other features, if present (Supplemental Fig. S2; Supplemental Table S1). MELT performs genotyping across all samples for both novel (non-REF) and reference (REF) mobile element copies to provide a comprehensive map of polymorphic MEIs in a given genome. MELT also evaluates the potential impact of each MEI on nearby genes and lists the gene features that are impacted (e.g., promoter, coding exon, intron, UTR, or terminator). Finally, we developed a quality tranche system that leverages the evidence at each MEI site to estimate the quality of the MEI breakpoint (Supplemental Table S2; Supplemental Methods). These tools and features are all included in the comprehensive MELT ver. 2.0 package, which has several improvements over the original MELT ver. 1.0 that was used for the 1000 Genomes Project (Supplemental Tables S1, S3; see below).

MELT ver. 2.0 was developed to work with diverse computational architectures and experimental designs. In this regard, several run modes were developed to provide flexibility in implementation, including (1) the single-sample (MELT-Single) mode, (2) the multiple-sample (MELT-Split) mode, where the four major steps of MELT are sequentially launched by the user, and (3) the multiple-sample (MELT-SGE) automated mode, where Sun Grid Engine (SGE) is used to automate the submission and processing of multiple samples (Supplemental Fig. S1). We also developed an Amazon Machine Image (AMI) version of MELT to facilitate MEI discovery in the cloud. The MELT-Single mode is useful for discovering and annotating MEIs in a relatively small number of genomes, whereas the MELT-Split and MELT-SGE multiple sample modes are engineered for population-scale studies involving hundreds or thousands of genomes.

As outlined above, an additional advantage of the multiple sample modes is that evidence for each MEI is drawn from multiple genomes (instead of just one) to identify the MEI site, associated features, and genotypes. To illustrate the increased sensitivity that is gained with the multiple sample MELT-SGE mode vs. the MELT-Single mode, we analyzed 10 low-coverage (6.0 to 17.0×) CEU whole genome sequences and five high-coverage (60×) whole genome sequences with both modes and compared the outcomes (see Supplemental Table S4 for the genomes that were analyzed; The 1000 Genomes Project Consortium 2015). The multiple-sample MELT-SGE mode clearly increases the sensitivity of MEI detection compared to the MELT-Single mode for all three MEI classes (*Alu*, L1, and SVA) (Supplemental Fig. S3).

### Scalability of MELT with Illumina WGS data

**Figure 1.**
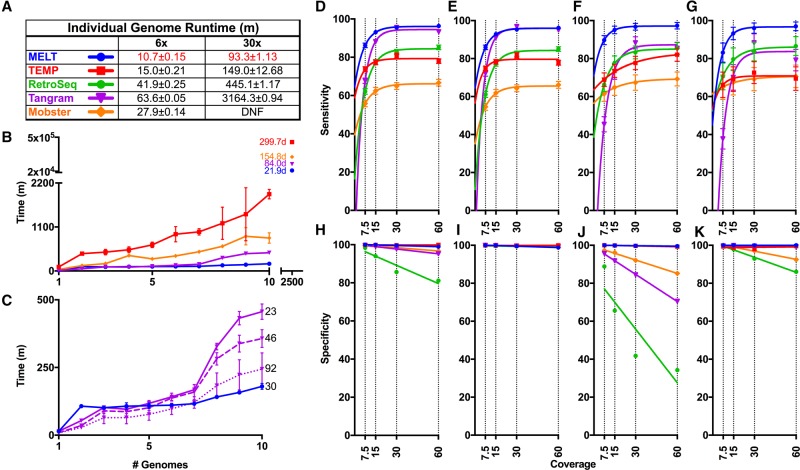
Comparisons of MEI discovery algorithms. (*A*–*C*) Runtime comparisons between MELT and four other MEI discovery algorithms: RetroSeq Mobster, Tangram, and TEMP. (*A*) Runtime in minutes on either a 6× or 30× coverage genome using a single processor (numbers are mean ± SD), with the best time for each coverage indicated in red. (*B*) Time required for each algorithm to analyze between one and 10 genomes using a distributed computing cluster. Shown to the *right* of experimental data are extrapolated estimates of the total runtimes for 2504 genomes with each algorithm. (*C*) Identical to *B* but depicting the median runtime for only MELT and Tangram. Tangram was run with 23, 46, or 92 threads (numbers to the *right* of lines). (*D*–*G*) Comparison of sensitivities for MELT and the MEI detection algorithms outlined above. False negative rates (FNRs) are plotted for (*D*) Aggregate, (*E*) *Alu*, (*F*) L1, and (*G*) SVA. (*H*–*K*) Comparison of specificities for MELT and the MEI detection algorithms outlined above. False discovery rates (FDRs) are plotted for (*H*) Aggregate, (*I*) *Alu*, (*J*) L1, and (*K*) SVA (Supplemental Table S5).

We next compared the clock speed and scalability of MELT ver. 2.0 with four existing MEI detection tools, i.e., TEMP (Zhuang et al. 2014), RetroSeq (Keane et al. 2013), Mobster (Thung et al. 2014), and Tangram (Wu et al. 2014). MELT ver. 2.0 had the fastest runtimes among the five MEI detection tools at both 6× and 30× coverages using Illumina WGS data from sample NA12878 (Fig. 1A; Supplemental Table S5; The 1000 Genomes Project Consortium 2015). We also compared the scalability of these tools using 10 low-coverage CEU genomes (Fig. 1B,C). For this test, we examined each tool for its ability to perform MEI discovery in one to 10 low-coverage (6.0 to 17.0×) CEU whole genome sequences (Supplemental Table S4; The 1000 Genomes Project Consortium 2015). Again, MELT had the best performance in these scalability tests (Fig. 1B,C; Supplemental Table S5).

By extrapolating these scalability curves to all 2504 low-coverage genomes that were sequenced by the 1000 Genomes Project, MELT was predicted to require 21.9 d to perform MEI discovery in all 2504 genomes (Fig. 1B, top). This estimate is in good agreement with the MELT runtimes that we actually observed when we processed all 2504 genomes for the 1000 Genomes Project on our computational cluster (∼2.5 wk). In contrast, Tangram was estimated to require 84 d (12 wk), Mobster 154.8 d (22.1 wk), and TEMP 299.7 d (42.8 wk) to complete MEI discovery in these genomes (Fig. 1B). Therefore, on the basis of these tests, MELT is estimated to perform population-scale MEI discovery ∼3.8-fold faster than Tangram, ∼7.1-fold faster than Mobster, and ∼13.7-fold faster than TEMP in head-to-head comparisons (Fig. 1B; Supplemental Table S5).

### Simulation studies to evaluate sensitivity and specificity

To evaluate the sensitivity and specificity of MELT ver. 2.0, we conducted a series of simulation studies (Fig. 1D–K; Supplemental Figs. S4, S5; Supplemental Tables S5, S6). Briefly, a test set of *Alu*, L1, and SVA MEIs was inserted randomly into the reference human genome multiple times to generate a series of simulated genomes containing new MEIs. We used similar sets of non-REF MEIs that were discovered in the NA12878 genome (*n* = 1114, including 922 *Alu*s, 146 L1s, 46 SVAs) (The 1000 Genomes Project Consortium 2015; Sudmant et al. 2015) but redistributed them randomly in the reference human genome and replicated this process 50 independent times (Methods; Supplemental Methods). We then generated FASTQ files from these genomes using an Illumina paired-end read simulator at 7.5×, 15×, 30×, and 60× coverage (Li and Durbin 2010). Finally, we mapped these reads to the reference human genome, generated BAM files, and tested MELT's ability to detect these artificially inserted MEIs. In head-to-head comparisons with TEMP, RetroSeq, Mobster, and Tangram, these simulations indicated that MELT had the best sensitivity and specificity curves for all three classes of MEIs over the range of simulated WGS coverages that were tested (Fig. 1D–K; Supplemental Table S5). Extensive PCR-based validations with sites selected from the 1000 Genomes Project data set (64 *Alu* sites, 54 L1 sites, and 59 SVA sites—a total of 177 sites that were discovered with MELT ver. 1.0) were in agreement with these MELT simulations (Sudmant et al. 2015). Likewise, an additional 90 PCR validations with MEI sites that were discovered with MELT ver. 2.0 also were in agreement with these simulations (Supplemental Methods; Supplemental Figs. S6, S7; Supplemental Table S7; see below). Overall, these benchmarking, simulation, and PCR validation tests indicate that MELT ver. 2.0 outperforms existing MEI discovery tools in terms of speed, scalability, sensitivity, and specificity, while also detecting a broader spectrum of MEI-associated features (Fig. 1; Supplemental Figs. S4–S7; Supplemental Tables S1, S5–S7).

### New 1000 Genomes Project and chimpanzee call sets

We next used the improved MELT ver. 2.0 package to rediscover *Alu*, L1, and SVA MEIs in the 2504 low-coverage and 30 high-coverage human genomes that were sequenced for Phase III of the 1000 Genomes Project. The resulting MEI call sets are more extensive than the original 1000 Genomes Project Phase III MEI call sets and include additional MEI features (Supplemental Fig. S6; Sudmant et al. 2015). Our new call set includes 6089 or 36.6% additional MEI calls, the majority of which are rare MEIs that were not detected in our previous 1000 Genomes Project call sets (Supplemental Fig. S6; Sudmant et al. 2015). MELT ver. 2.0 employs slightly different rules for using DRPs and SRs compared to MELT ver. 1.0 (Methods), and these changes led to the improved detection of rare MEIs while retaining similar overall results in benchmarking and validation tests (Fig. 1; Supplemental Figs. S6, S7; Supplemental Tables S5–S7). The *Alu*, L1, and SVA MEIs that we discovered with MELT ver. 2.0 had frequency distribution curves that were remarkably similar to the SNP frequency curve that was generated for the same samples by the 1000 Genomes Project (Supplemental Fig. S6). In contrast, the MEIs that were called with MELT ver. 1.0 did not resemble this SNP curve as closely and lacked sensitivity in the lowest allele frequency bin (labeled 0.00) (Supplemental Fig. S6).

We also adapted MELT ver. 2.0 to perform MEI discovery in chimpanzees. MELT was designed to be flexible in this regard, and the requirements for adapting MELT to a new species are fairly minimal: (1) A reference genome sequence must be available for the species; and (2) a set of reference endogenous MEI sequences must be available (or generated) for the species. Both of these requirements were met for chimpanzees, and thus, we used MELT to perform MEI discovery on 25 chimpanzees whose genomes had been sequenced previously (Prado-Martinez et al. 2013). Our chimpanzee MEI data set includes 7278 *Alu* and 4,381 L1 MEIs (Supplemental Fig. S8). These chimpanzee data, and similar data sets generated with other organisms including canines (EJ Gardner and SE Devine, unpubl.), demonstrate that MELT can be readily adapted to other organisms.

### Interior mutations and subfamily analysis

Mutations are encountered frequently within the interior sequences of MEIs due to the error-prone L1 reverse transcriptase that replicates *Alu*, L1, and SVA elements (Gilbert et al. 2005). These mutations (and patterns of mutations) have been useful for determining whether a given MEI belongs to a lineage that is known to be active in humans (Boissinot et al. 2000; Batzer and Deininger 2002; Wang et al. 2005; Konkel et al. 2015). Thus, we developed new MELT tools to identify interior mutations in MEIs and assign MEIs to lineages (or subfamilies). Since *Alu* and L1 MEIs together represent 95.3% of the MEIs that were discovered in the 1000 Genomes Project, we initially focused on developing tools for these two element classes. Specifically, we developed a tool named C*Alu* that identifies interior mutations within *Alu* elements and then uses these mutations to assign *Alu*s to known subfamilies and a similar tool named LINEu that carries out comparable functions for L1 elements. These tools were validated in the simulation studies outlined above (Supplemental Fig. S4) and then used to identify interior mutations in the updated 1000 Genomes Project call set. The resulting subfamily analysis revealed that the distributions of active *Alu* and L1 MEIs in the 1000 Genomes Project data sets were similar to those observed in previous studies in humans, thus providing additional validation for our methods. For example, *Alu*Ya5, and *Alu*Yb8 elements, which are known to be the most abundant *Alu* subfamilies in humans (Batzer and Deininger 2002; Bennett et al. 2008), also were the most abundant *Alu* MEIs that we discovered in the updated 1000 Genomes Project data set (Supplemental Fig. S9). Likewise, extensive testing of LINEu on fully sequenced FL-L1 (full-length L1) elements indicated that LINEu accurately identifies known human-specific L1 subfamilies (L1-Ta0, L1-Ta1, L1-Ta1d, and L1-Ta1nd) (Scott et al. 2016).

### Stratification of MEIs in 1000 Genomes Project populations

**Figure 2.**
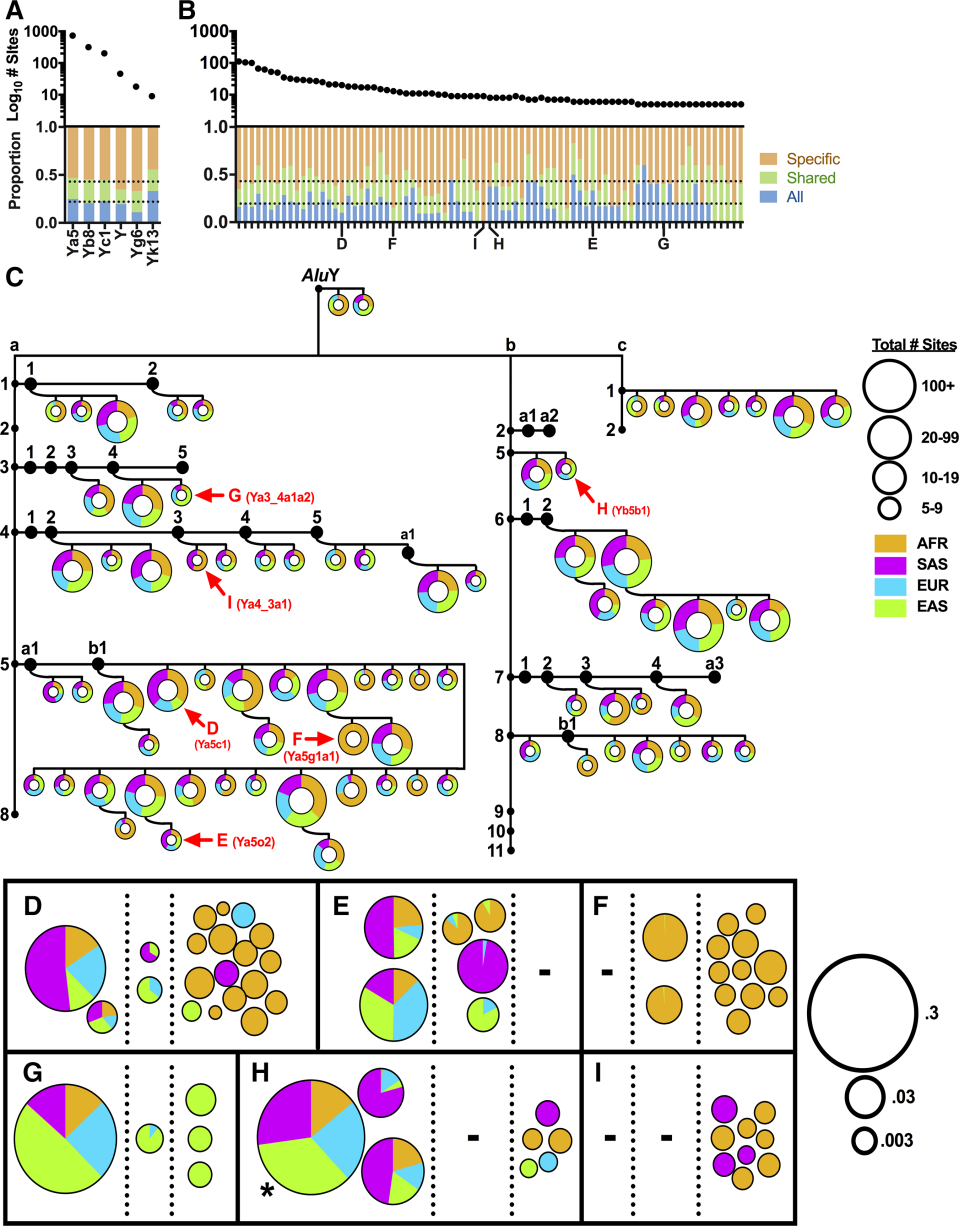
Complex patterns of *Alu* subfamily expansion in diverse human populations. Six known *Alu* subfamilies (*A*) and 79 novel *Alu* subfamilies (*B*) that were identified using interior sequence changes were analyzed for sharing among the 1000 Genomes Project nonadmixed continental populations. Plotted are: (*top*) log_10_ total sites in each subfamily; (*bottom*) proportion of sites shared among all continental populations (blue); proportion of sites shared by two or three continental populations (green); proportion of sites that are specific to one continental population (brown). The average proportion for each category is indicated by a horizontal dotted line. (*C*) Tree of 79 novel *Alu*Y subfamilies. We required at least five independent copies with a novel set of interior mutations (excluding CpG sites) to establish new subfamilies. This threshold is fairly conservative and eliminates errors introduced by Illumina sequencing. After C*Alu* classification (Supplemental Fig. S9), novel *Alu* subfamilies were placed on a tree of known *Alu*Y families (a, b, and c shown) and subfamilies (small black circles). Each pie chart represents the sum of allele counts for all constituent sites of a particular novel subfamily with the total number of identical loci represented by the diameter of the pie (see Supplemental Fig. S10 for figure key). (AFR) African; (SAS) South Asian; (EUR) European; (EAS) East Asian. (*D*–*I*) Families with unique population sharing are shown, with each pie representing the proportion of total alleles from each of the four major continental populations of the 1000 Genomes Project. Each site is placed into one of three categories based on population sharing: present in all four continental populations (*left*); in two or three continental populations (*middle*); or in one continental population (*right*). Pies are sized based on the log_10_ allele frequency of each site. (*) Actual AF is 0.52446. *Alu* subfamilies were named as outlined in Batzer et al. 1996 (Supplemental Table S8).

We next examined the population stratification of MEIs in the updated call sets that we generated from the 1000 Genomes Project. Varying degrees of *Alu*, L1, and SVA sharing were observed across the four major continental groups of the 1000 Genomes Project. *Alu* subfamilies such as *Alu*Ya5, *Alu*Yb8, *Alu*Yc1, *Alu*Y, *Alu*Yg6, and *Alu*Yk13 included copies that were shared by all nonadmixed continental groups, as well as those that were found in a subset of groups or in a single group (Fig. 2A). None of these copies was found in the chimpanzee data set. These data indicate that several major human-specific *Alu* subfamilies have been active for most of modern human history, since these subfamilies have generated some MEI loci that are sufficiently old to be found in all modern humans, while generating other loci that are sufficiently young to be restricted to a single continental group or subpopulation.

We also examined the distributions of 79 novel *Alu* subfamilies that we identified in the updated 1000 Genomes Project MEIs through analysis of shared interior mutation patterns (Fig. 2B,C; Supplemental Table S8; Supplemental Fig. S10). Although some of these novel *Alu* subfamilies were found in all four of the major continental groups of modern humans, others had very unequal distributions. For example, Family F is mostly restricted to the AFR continental population, whereas Family G is enriched in the EAS continental population (Fig. 2B,C). Many of these very young *Alu* subfamilies have unique blends of sharing, and a sampling of the spectrum of sharing is depicted in Figure 2, D through I. For example, Family I includes copies that are shared only by AFR and SAS individuals, suggesting that these copies might have been influenced by population bottlenecks during migration out of Africa (OOA) or by admixture. Overall, these data reveal complex patterns of *Alu* subfamily stratification in the 1000 Genomes Project populations, likely reflecting diverse patterns of demographic histories and other population forces affecting MEI dynamics (see below).

### Active full-length L1 source elements in human populations

**Figure 3.**
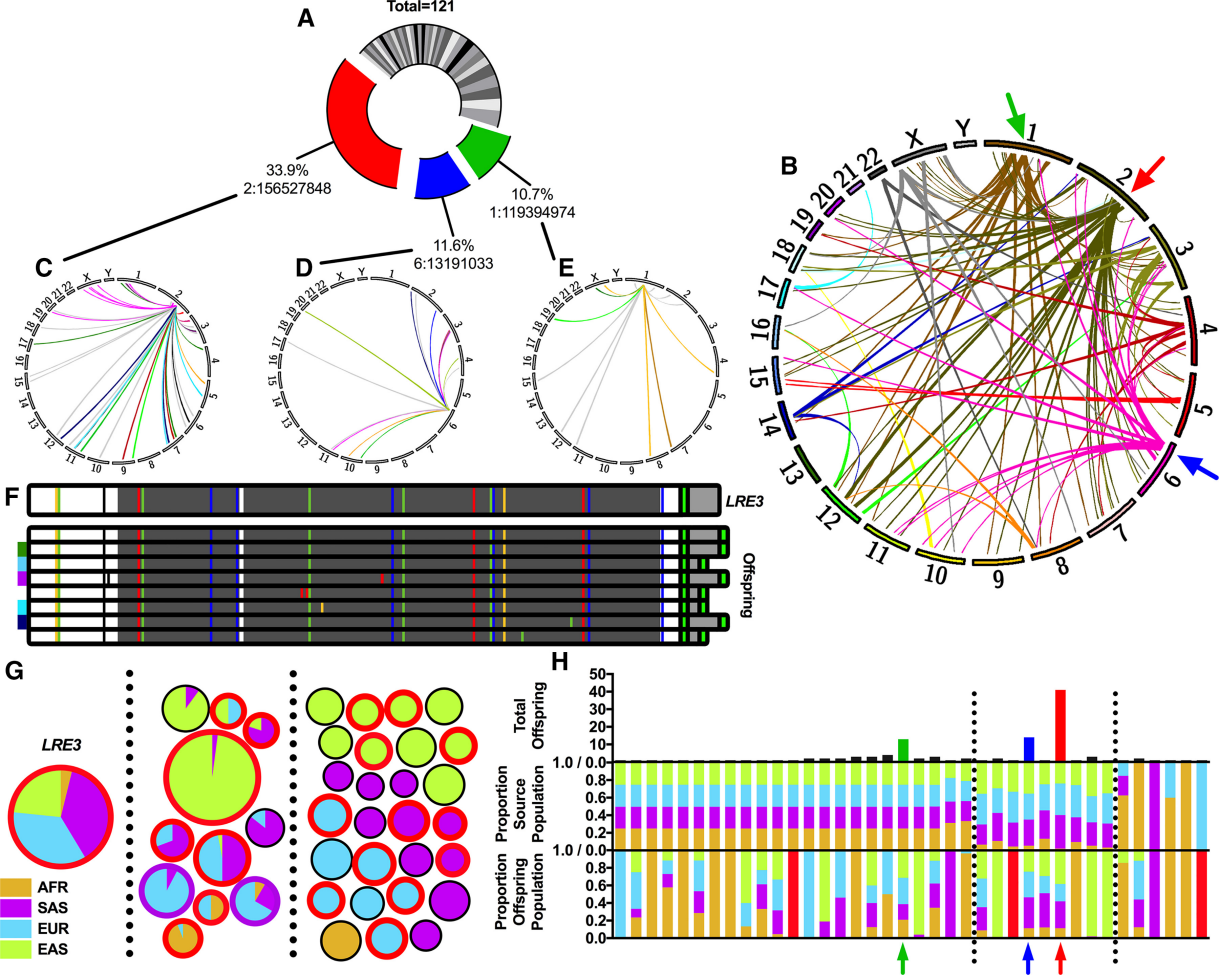
Analysis of L1 source-offspring relationships using 3′ transductions. (*A*) Pie chart depicting the proportion of offspring attributable to each of the 38 FL-L1 source elements identified in this study. One hundred twenty-one out of 4118 (2.9%) of the L1s identified had 3′ transductions that could be used to identify the FL-L1 source elements that produced these offspring insertions (Supplemental Table S9). Note that this method can only be used to track source/offspring relationships for L1′s that produce 3′ transductions. The *LRE3*, Chr 6: 13191033, and Chr 1: 119394974 FL-L1 source elements are indicated in red, blue, and green, respectively. (*B*) Circos plot (Krzywinski et al. 2009) depicting the genomic landscape of source-offspring relationships summarized in *A*. Red, blue, and green arrows indicate the three FL-L1 source elements highlighted in *A*. (*C*–*E*) Individual Circos plots tracking offspring for the three most active FL-L1 source elements from *A*. Each source-offspring relationship is colored based on the population in which the offspring element is found (gray if found in multiple populations). (*F*) *LRE3* was sequenced from an individual of European descent (*top*), along with eight FL-L1 *LRE3* offspring. Sequence changes compared to the L1.3 FL-L1 element (Dombroski et al. 1993) are shown as blue, green, yellow, red, or black vertical lines representing C, A, G, T, or deletion mutations, respectively. All eight sequenced FL-L1 offspring of *LRE3* have two intact ORFs (dark gray bars). The first poly(A) tail is shown in bright green, with transduced sequence shown in light gray. Offspring elements that have a 3′ transduction also have a second poly(A) tail (bright green). The five population-specific FL-L1 elements are indicated by the 1000 Genomes Project population colors next to the elements. (*G*) *LRE3* transduction family, displayed in a similar manner to Figure 2 (American [AMR]-specific offspring not shown; *n* = 3). Each pie chart represents either *LRE3* (labeled) or one *LRE3* offspring locus from *C*. Borders of each pie are colored red if the element is a FL-L1 (*n* = 20), or purple if it has a 5′ inversion (*n* = 2). (*H*) Source and offspring element population distributions. Shown for each source element is the total number of offspring (*top*), the population distribution of the source element (*middle*), and the aggregate population distribution of all offspring (*bottom*). Highlighted with colored arrows are source elements from *A*. Red bars indicate where offspring were only found in the American continental population. Vertical black dotted lines separate source elements into one of three classes: found in all populations (*left*), found predominantly in OOA populations (*middle*), and found in a subset of other populations (*right*).

We also developed two new tools to identify FL-L1 source elements that recently have generated MEI offspring in humans. One of these tools leverages 3′ transduction events (Fig. 3), and the other leverages interior mutation profiles, to track source/offspring relationships (Scott et al. 2016). A 3′ transduction event occurs when a short segment of adjacent genomic sequence is incorporated into an offspring MEI during retrotransposition (Moran et al. 1999). A 3′ transduction is initiated at the level of transcription: Transcripts originating from a FL-L1 source element bypass a weak poly(A) signal at the 3′ end of the element and instead use an alternative poly(A) signal that is encountered in the adjacent downstream genomic region. When the resulting chimeric FL-L1 transcript is used as a replication template during retrotransposition, 3′ adjacent genomic sequences are replicated and mobilized along with the L1 source element. These 3′ transductions can be used to track source/offspring relationships because they serve as unique address tags that are associated with a single L1 source element and its offspring (Moran et al. 1999; Tubio et al. 2014). Thus, we developed a new tool to study source/offspring relationships using 3′ transductions and validated it with simulations (Supplemental Methods; Supplemental Fig. S4F; Supplemental Table S6).

By applying this 3′ transduction tool to the 2504 low-coverage human genomes that were sequenced by the 1000 Genomes Project, we identified 121 L1 offspring insertions that carried 3′ transductions (Fig. 3). We then used these unique 3′ transduction tags to identify the 38 FL-L1 source elements that produced these insertions. Three of the 38 FL-L1 source elements, the Chr 2: 156527848, Chr 6: 13191033, and Chr 1: 119394974 elements, were exceptionally active and collectively generated more than half (68/121 or 56.2%) of the offspring MEIs (Fig. 3A–E). The most active element among these FL-L1 elements, the Chr 2: 156527848 element, is a previously identified “hot L1” source element known as *L1RE3* (also known as *LRE3*) (Brouha et al. 2002), and it alone generated 41/121 (33.9%) of the offspring insertions (Fig. 3A–C). The Chr 6: 13191033 and Chr 1: 119394974 FL-L1 elements generated an additional 14 and 13 offspring, respectively (Fig. 3A,B,D,E), while the remaining 35 FL-L1 source elements each generated between one and four offspring (Fig. 3A,B; Supplemental Table S9).

We found that the three highly active FL-L1 source elements (i.e., *LRE3*, Chr 6: 13191033, and Chr 1: 119394974) had diverse patterns of stratification among the 1000 Genomes Project populations. *LRE3* is clearly enriched in OOA populations (Fig. 3G,H) and has very low allelic frequencies in AFR populations (with MAFs ranging from 0 to 0.025 in the six AFR subpopulations). Likewise, offspring insertions produced by *LRE3* followed a similar pattern of enrichment in OOA populations, with the majority of offspring localized to one or more OOA populations (Fig. 3G,H). *LRE3* itself has generated at least 20 FL-L1 offspring insertions that could, in principle, serve as new FL-L1 source elements (Fig. 3F,G). We fully sequenced eight of these 20 FL-L1s (Fig. 3F), and all eight had two intact open reading frames (ORFs), providing further support that they might serve as active source elements. Interestingly, five of these eight FL-L1 insertions were found in only one OOA population (Fig. 3F). The Chr 6: 13191033 FL-L1 source element and its offspring had population distributions that were very similar to *LRE3*, whereas the Chr 1: 119394974 FL-L1 source element and its offspring were more evenly distributed among the four continental groups (Fig. 3H). These correlated patterns of FL-L1 source elements and their offspring suggest that L1 mutagenesis is influenced by the stratification of these highly active source elements. Moreover, the birth of new source elements within these lineages might further enhance L1 mutagenesis in these lineages over time.

The remaining 35 FL-L1 elements that produced 3′ transductions fell into three categories: (1) source elements that were shared almost equally across the four continental populations (Fig. 3H, left, middle panel); (2) those that were shared unequally by the four continental populations (Fig. 3H, center, middle panel); and (3) those that were found in less than four (between one and three) continental populations (Fig. 3H, right, middle panel). Unlike the three highly active elements described above (*LRE3*, Chr 6: 13191033, and Chr 1: 119394974), which had fairly correlated patterns of source and offspring distributions, the offspring patterns for these remaining 35 FL-L1 source elements were very diverse and often were not correlated with the distributions of their respective source elements (Fig. 3H, lower panel). For example, even though some FL-L1 elements were present in all four continental groups (Fig. 3H, center, middle panel), the offspring from these elements often were found in only one or two continental populations (Fig. 3H, center, lower panel). In some cases, this might be due to an ascertainment bias caused by the small number of offspring that were produced by these elements. However, it is also possible that some of these FL-L1 source elements are active in a subset of the populations in which they reside. Overall, these data reveal diverse relationships of FL-L1 source elements and their offspring in modern human populations.

The complete interior sequences of the FL-L1 source elements that produced 3′ transductions in our study were obtained for 34/38 (89.5%) of the elements (either from the reference genome or from PacBio sequencing) (Supplemental Methods; Supplemental Table S9). Most of the sequenced elements (25/34 or 73.5%) have two intact ORFs and belong to one of four active L1Ta subfamilies (Ta0, Ta1, Ta1d, Ta1nd). Many of these elements also have been found to be active in a cell culture-based assay for L1 retrotransposition (Brouha et al. 2002, 2003; Beck et al. 2010). Thus, at least some of these elements are likely to have retained the ability to produce MEIs in humans. The remaining 9/34 (26.5%) of the sequenced source elements had only one or zero intact ORFs and thus appear to have accumulated deleterious mutations that have rendered them inactive. These include one PA2 element, four Ta elements (Ta0, Ta1, Ta1d, Ta1nd), and one noncanonical L1 element. Our analysis also revealed a range of poly(A) signal configurations for these FL-L1 elements, suggesting that the underlying reasons for the production of 3′ transductions are complex (Supplemental Discussion; Supplemental Table S9).

### Comparisons with L1 source elements that are somatically active in cancer genomes

**Figure 4.**
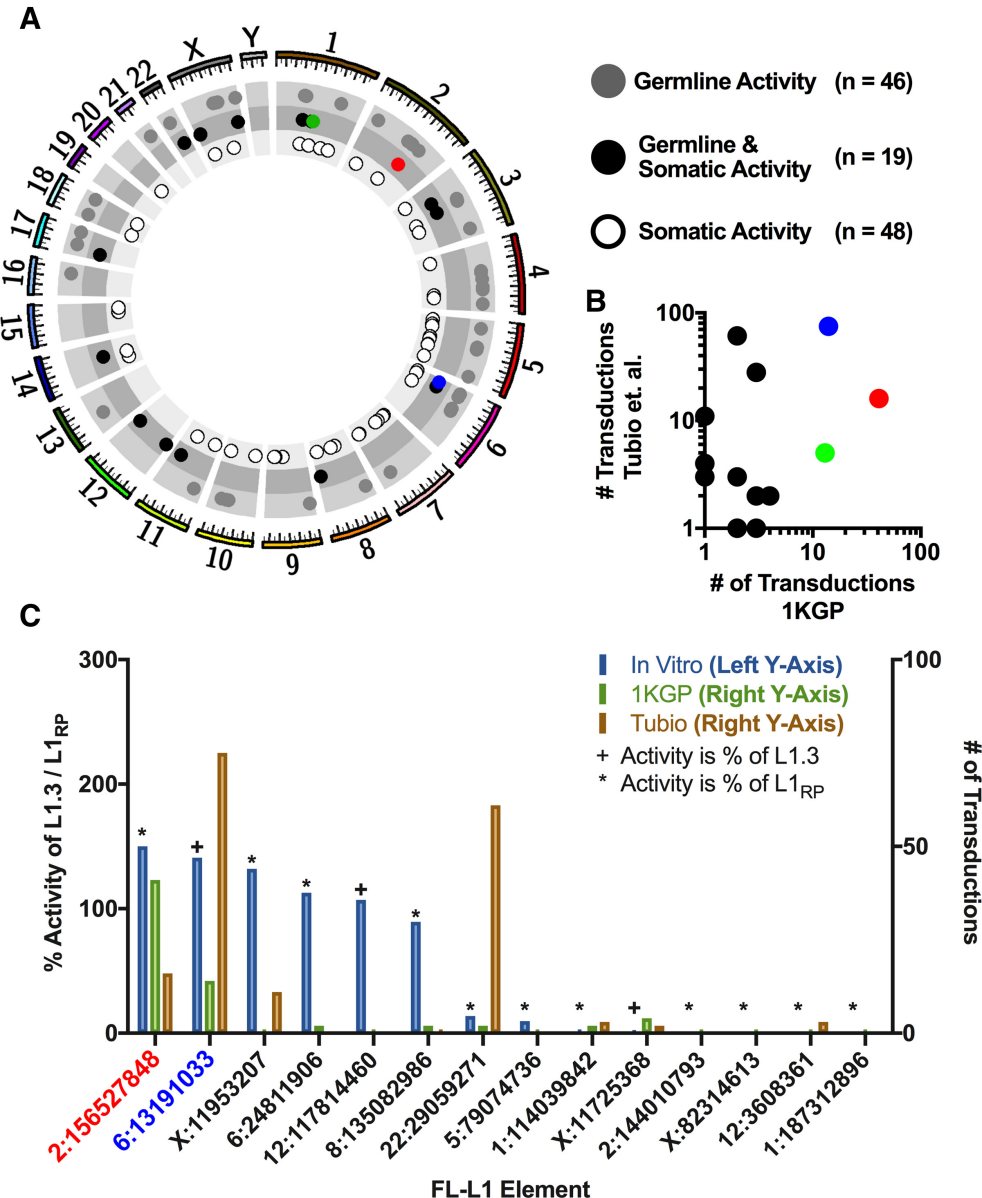
Recently-active FL-L1 source elements in human populations and cancers. (*A*) Circos plot of the human genome with coordinates of known active FL-L1 source elements producing 3′ transductions (circles) (Supplemental Table S9). FL-L1s are further separated into one of three categories based on the tissue type(s) in which activity was recorded. The three most active FL-L1 source elements identified in this study are represented as circles corresponding to the colors in Figure 3A. (*B*) Log-log plot depicting 14 L1 source elements that were active in the germline (this study) and somatic tumors (Tubio et al. 2014). The three most active germline elements (this study) are highlighted according to the colors in Figure 3A and are active in somatic cancers as well. Note that one of the dots represents two separate L1 source elements, as both have the same number of germline and somatic offspring. (*C*) Comparison of FL-L1 element activity in the cell culture-based retrotransposition assay (percent of L1.3 or L1RP activity, light blue) (Brouha et al. 2002, 2003; Beck et al. 2010) with the total number of offspring identified in this study (light green) and the Tubio et al. study (light orange) (Tubio et al. 2014). Only elements that were active in the germline (this study), cancers (Tubio et al. 2014), and the cell culture-based assay were displayed.

We next compared the active FL-L1 source elements that we identified through 3′ transductions in the 1000 Genomes Project samples (Fig. 3) with those that had been reported previously in the literature (Supplemental Table S9). A total of 113 nonredundant FL-L1 source elements were identified in these collective studies (including our study) (Fig. 4A). Forty-six out of 113 (40.7%) of these FL-L1 source elements were active exclusively in the germline, whereas 48/113 (42.5%) were active exclusively in somatic cancer tissues (Fig. 4A; Supplemental Fig. S11). Another 19/113 (16.8%) were active in both the germline and somatic tissues (Fig. 4A). The three most active FL-L1 source elements that we identified in our study also were among the most active elements in a large-scale somatic cancer study (Fig. 4B; Tubio et al. 2014). Thus, some FL-L1 source elements are highly active in both the germline and somatic tissues, whereas others are active in only one of these tissue types.

We also compared these germline and somatic FL-L1 source element activities (Fig. 4) with those that had been measured previously in a cell culture-based assay for L1 retrotransposition (Fig. 4C; Moran et al. 1996; Brouha et al. 2002, 2003; Beck et al. 2010). The *LRE3* FL-L1 source element, which is highly active in both the germline and somatic cancer tissues, is also highly active in the cell culture-based retrotransposition assay (Fig. 4C; Brouha et al. 2002; Beck et al. 2010). Although several other FL-L1 source elements had similarly correlated levels of retrotransposition in the germline, somatic tissues, and cultured cells, other elements behaved quite differently in these three cellular environments. For example, the Chr 22: 29059271 FL-L1 source element is highly active in somatic cancer genomes but is relatively inactive in both the germline and the cell culture-based assay (Fig. 4C; Brouha et al. 2003; Tubio et al. 2014). Other discordant patterns of activity also were observed among these tissues and assays (Fig. 4C). Overall, these data indicate that a given FL-L1 source element can have remarkably different levels of activity in these three cellular environments.

### 5′ inversions

L1 elements often produce MEI offspring that have 5′ inversions (i.e., the 5′ portion of the MEI is inverted relative to the 3′ portion). These 5′ inversions have been proposed to be caused by a mechanism termed “twin priming,” whereby the TPRT process is initiated simultaneously from both strands of DNA at the genomic integration site (Ostertag and Kazazian 2001). Another feature of these 5′ inverted L1 MEIs is that they often have small insertions, deletions, or duplications at the inversion junctions. However, these MEIs otherwise appear to have all of the typical features of L1 insertions, including poly(A) tails, TSDs, and the other features outlined in Supplemental Table S1.

**Figure 5.**
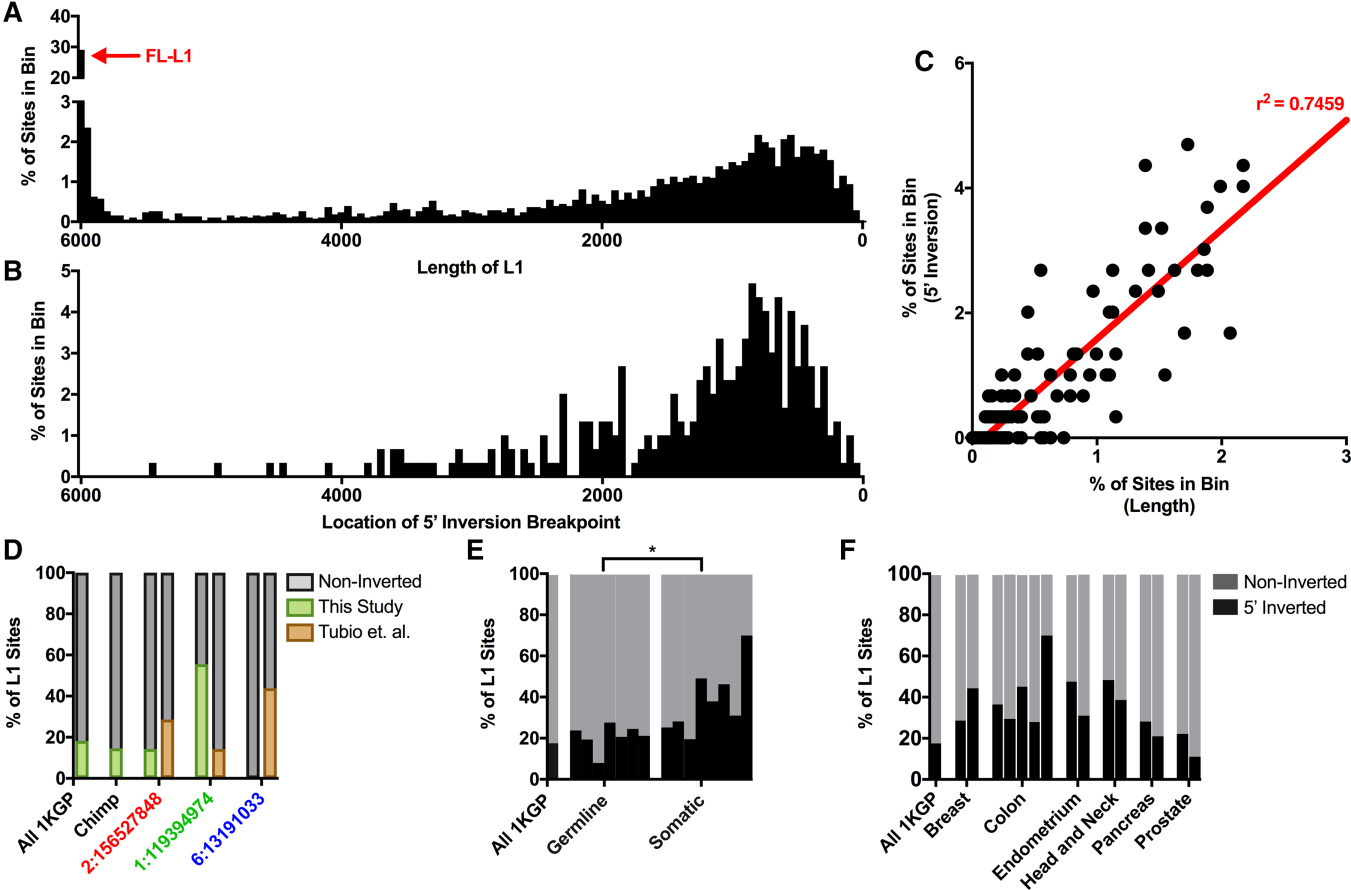
L1 5′ inversions in germline and somatic tissues. (*A*) L1 length distribution among sites discovered in the 1000 Genomes Project Phase III samples. (*B*) 5′ inversion positions discovered in the 1000 Genomes Project Phase III samples (Supplemental Table S10). (*C*) Correlation between the distributions shown in *A* and *B*, with the linear trend line and r^2^ correlation shown in red. FL-L1 (red arrow in *A*) sites were excluded from this comparison because no correlation was observed in the first ∼590 bp of FL-L1 elements. (*D*) 5′ inversion rates among all L1 sites in the 1000 Genomes Project, chimpanzee, and among particularly active 3′ transducers highlighted in Figure 3, C–E. (*E*) Total number of 5′ inverted sites from 1000 Genomes Project MEIs (this study) compared with other germline and somatic studies. The proportion of 5′ inverted sites is significantly different ([*] *P* = 0.0207) between germline and somatic insertions (Supplemental Table S10). (*F*) Comparison of germline 5′ inversion rates (1000 Genomes Project MEIs) and several different tumor types analyzed by various studies (Supplemental Table S10).

Because 5′ inversions occur frequently, we developed a new tool to identify 5′ inversions in L1 offspring elements and validated it in simulation studies (Supplemental Fig. S4G,H; Supplemental Table S6). We then applied this tool to the 1000 Genomes Project samples and found that 298/1634 (18.2%) of the L1 MEIs discovered in the 1000 Genomes Project samples had 5′ inversions (Note: in some cases we could not measure 5′ inversions due to a lack of DRP/SR evidence at one end of the L1 MEI). The inversion junctions for these 298 non-REF MEIs generally were located throughout the reference L1 sequence as reported previously for older REF L1 elements (Fig. 5A,B; Szak et al. 2002). However, we noted a depletion of 5′ inversions near the 5′ end of L1. In fact, we did not identify a single 5′ inversion junction in the first 590 bp of L1, despite the fact that 33.8% of the non-REF L1 MEIs discovered in the 1000 Genomes samples were either full-length or otherwise contained sequences that spanned this region (Fig. 5A,B). We verified that MELT had sufficient sensitivity in the first 590 bp to detect 5′ inversions in this region (Supplemental Fig. S4H; Supplemental Methods). These data suggest the possibility that the 5′ inversion mechanism requires ∼500 bp of free RNA or DNA at the 5′ end of L1, perhaps to loop back and serve as a priming site for DNA replication.

We also examined the rates at which 5′ inversions are produced from diverse FL-L1 source elements and whether these rates vary from one FL-L1 source element to the next. To explore this question, we examined the 5′ inversion rates for the FL-L1 source elements that were associated with 3′ transductions in the 1000 Genomes Project data set (Fig. 3). Interestingly, the three most active FL-L1 source elements from this data set (i.e., *LRE3*, Chr 6: 13191033, and Chr 1: 119394974) (Fig. 3) generated 5′ inversions at very different rates (i.e., 55.6% of offspring had 5′ inversions for the Chr 1: 119394974 FL-L1 source element, 14.3% for the *LRE3* FL-L1 element, and 0.0% for the Chr 6: 13191033 FL-L1 element) (Fig. 5D; Supplemental Table S10). We also examined the 5′ inversion rates in several additional studies involving germline and somatic MEIs and likewise observed a range of 5′ inversion rates in these studies (Fig. 5D–F; Supplemental Table S10). These data suggest that the rates at which FL-L1 source elements produce 5′ inversions may vary among FL-L1 source elements and across diverse cellular environments.

### Ancient MEIs in Neanderthal and Denisovan genomes

**Figure 6.**
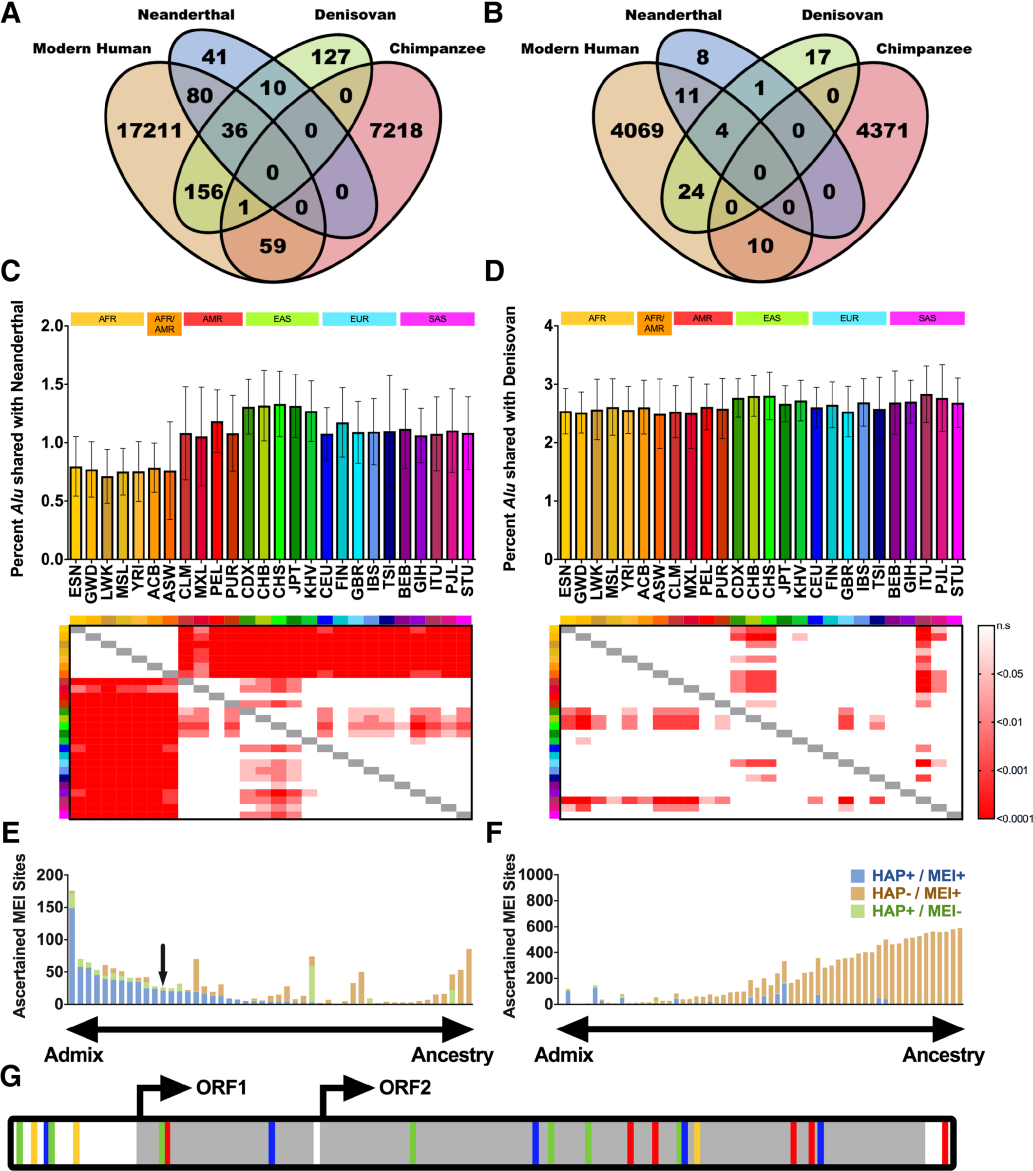
Mobile element activity in ancient human genomes and introgression of ancient MEIs into modern humans. (*A*,*B*) Sharing of (*A*) *Alu* and (*B*) L1 MEIs between Neanderthal, Denisovan, modern humans, and chimpanzees (Supplemental Table S11). (*C*,*D*) Sharing of Neanderthal and Denisovan *Alu* MEIs in each of the 26 1000 Genomes Project Phase III populations. For each population, we determined the average percentage per individual of *Alu* MEIs shared with (*C*) Neanderthal or (*D*) Denisovan. Heat maps represent multiple comparison ANOVA *P*-values between each population (key at *right*). (*E*) Analysis of Neanderthal MEI introgression in non-African individuals. Each bar represents one MEI site that was shared between Neanderthal and non-African individuals (i.e., the site was found only in SAS, EUR, and/or EAS). Bars are colored by MEI overlap with Neanderthal haplotypes (Supplemental Methods; Sankararaman et al. 2014), with sites to the *left* of the chart likely contributed to modern humans by introgression from Neanderthals and sites to the *right* likely due to common ancestry. “HAP” indicates whether the Neanderthal haplotype is present at the site (HAP+ or HAP−). “MEI” indicates whether the MEI is present at the site (MEI+ or MEI−). Blue bars indicate a high degree of linkage disequilibrium (LD) between the Neanderthal haplotype and the MEI (HAP+/MEI+), and brown bars indicate little or no correlation between the Neanderthal haplotype and the MEI (HAP-/MEI+). Blue sites have *r*^2^ values for HAP+/MEI+ of >0.5, whereas brown sites segregate independently (Supplemental Table S11; Supplemental Methods). The black arrow indicates the FL-L1 element described in *G*. (*F*) Analysis of Neanderthal MEI introgression in all individuals. Identical analysis to *E* but for sites with an AFR allele frequency greater than zero. (*G*) Cartoon of an FL-L1 element sequenced from a GBR individual, with differences shown as in Figure 3F. The quality of ancient MEI calls was comparable to those called in modern humans (Supplemental Fig. S12).

Ancient genomes from Neanderthals and Denisovans have been technically challenging to sequence and analyze, and thus far no MEIs have been successfully discovered in these archaic hominids. We next determined whether MELT could detect *Alu*, L1, and SVA MEIs in these genomes. Indeed, MELT successfully detected 41 ancient *Alu* MEIs in Neanderthals and 127 ancient *Alu* MEIs in Denisovans that were not found in chimpanzees or modern humans (Fig. 6A). We also discovered another 10 ancient *Alu* MEIs that were shared by Neanderthals and Denisovans but were absent from chimpanzees and modern humans (Fig. 6A). Similarly, 26 ancient L1 insertions and three ancient SVA insertions were identified in Neanderthals and Denisovans that were absent from chimpanzees and modern humans (Fig. 6B; Supplemental Table S11). Thus, *Alu*, L1, and SVA elements appear to have been active in ancient hominids during the period when they were temporally and geographically separated from modern humans (∼86,000 to 800,000 yr ago) (Sankararaman et al. 2014; Vattathil and Akey 2015).

We also identified 272 *Alu*, 39 L1, and 13 SVA elements that were shared by ancient hominids and modern humans but were absent from chimpanzees (Fig. 6A–D; Supplemental Table S11). In some cases, these shared MEIs likely were generated in a common ancestor prior to the migration of ancient hominids and modern humans out of Africa. However, 49 MEIs (42 *Alu* and 7 L1 MEIs) were shared exclusively between ancient hominids and modern OOA populations (i.e., they were absent from chimpanzee and AFR populations) (Fig. 6E; Supplemental Table S11). This class of sharing suggested the possibility that at least some of these MEIs initially were generated in ancient hominid genomes and then moved into modern human genomes through introgression during periods when ancient and modern humans cohabitated Europe and Asia. Indeed, archaic SNP haplotype maps (Sankararaman et al. 2014) indicated that these MEIs were almost exclusively embedded within Neanderthal and Denisovan haplotypes, supporting the idea that these ancient MEIs were integrated in the context of ancient genomes and then migrated into modern humans through introgression (Fig. 6E). Among these introgressed MEIs, we identified a FL-L1 element that might have been active in both ancient and modern humans (Fig. 6G; see Discussion). These MEI introgression data are in agreement with SNP-based models of introgression, whereby Neanderthal introgression is observed in all OOA modern populations (Fig. 6C) and Denisovan introgression is found mainly in East Asian populations (Fig. 6D). Thus, introgression of ancient *Alu*, L1, and SVA MEIs into modern human genomes is yet another mechanism of MEI dynamics in humans.

## Discussion

We have developed the MELT package of computational tools to efficiently discover and study MEIs in WGS projects. In head-to-head tests, we found that MELT outperformed existing MEI discovery tools in terms of speed, scalability, sensitivity, and specificity, while also detecting a broader range of MEI-associated features (Fig. 1; Supplemental Table S1). In addition to the basic MELT discovery algorithm, we also developed a set of companion tools to study the MEIs that are discovered by MELT (Supplemental Table S1). We likewise developed several run modes to improve the portability of MELT and to provide flexibility in experimental design. In addition to using MELT with the 1000 Genomes Project samples (Supplemental Fig. S6; Sudmant et al. 2015), we also have used it to discover MEIs in chimpanzees, ancient Neanderthal and Denisovan genomes (Fig. 6), cancer genomes (Scott et al. 2016), and canines (EJ Gardner and SE Devine, unpubl.). Thus, in principle, MELT could be used with any species or experimental design, provided that a reference genome sequence and a set of reference mobile element sequences are available for the organism of interest.

### Population dynamics of MEIs

Our study revealed extensive MEI stratification across diverse human populations. For example, the active FL-L1 source elements that we identified through 3′ transductions often were unequally distributed among the major continental groups of modern humans, and this appears to have led to differences in the production of L1 offspring in some cases (Fig. 3). Since FL-L1 source elements also are responsible for generating *Alu* and SVA MEIs, a highly active population-specific source element could have a major impact on the stratification of these other two classes of MEIs as well. In some cases, MEIs were shared by all continental groups and ancient hominids but were absent from chimpanzees, indicating that the MEI was generated very early in human history and is shared throughout the human and hominid lineages as a consequence of common ancestry. In other cases, MEIs were found only in ancient Neanderthal or Denisovan genomes or were restricted to AFR or OOA populations, suggesting that they were generated more recently. However, the complexity of sharing that we observed suggests that other forces such as admixture and bottlenecks likely have influenced the stratification of these elements as well. Our data provide an extensive map of MEIs extending from chimpanzees, ancient hominids, and modern humans and document these diverse MEI sharing patterns on the largest scale that has been examined to date.

We also noted a novel process that affected the population dynamics of MEIs: the introgression of ancient MEIs from Neanderthals and Denisovans into modern humans. Our data indicate that the mobilome of modern humans has been shaped at least partly by ancient *Alu* and L1 (and likely SVA) elements that initially were generated in archaic genomes and subsequently were introduced into modern humans through introgression. Ancient Neanderthals and Denisovans were exposed to harsh selective pressures in the face of new environmental challenges (Vattathil and Akey 2015), and some ancient MEIs could, in theory, have produced adaptive changes in these hominids. Such MEIs could, in turn, have been rapidly passed into modern humans through introgression, which could have conferred a selective advantage to recipients as they faced the same challenges. Thus, ancient MEIs that predated modern humans could have helped to influence modern human phenotypes through novel genetic mechanisms. As additional Neanderthal and Denisovan genomes are sequenced, it should be possible to further explore this possibility.

We also identified a FL-L1 element among the introgressed MEIs that might have served as an active source element in both ancient hominids and modern humans (Fig. 6G). This element was fully sequenced from a modern human genome using a PacBio-based approach that we developed recently (Scott et al. 2016). We found that the element is 6015 bp in length, has two intact ORFs, and belongs to the L1-Ta1d subfamily, which is one of the most active human-specific L1 subfamilies (Supplemental Table S9). The same L1-Ta1d subfamily gave rise to the highly active *LRE3* FL-L1 element (Figs. 3, 4) and other “hot” L1 source elements that have caused human diseases (e.g., see Scott et al. 2016). These data indicate that the L1-Ta1d subfamily originated sufficiently early to be present in ancient Neanderthal and modern human genomes, suggesting that this highly active subfamily was generated in a common ancestor at least ∼800,000 yr ago. Since the L1-Ta1d subfamily has generated a number of diseases in modern humans, it likely caused diseases in ancient hominids as well.

### Recently-active FL-L1 source elements identified with 3′ transductions

Our 3′ transduction tracking data provide evidence for many novel FL-L1 source elements that have been active recently in modern humans. Although we identified a total of 38 active FL-L1 source elements in our studies, just three of these elements produced the majority of germline insertions that are associated with 3′ transductions in the 1000 Genomes Project populations (Figs. 3, 4). Many of the FL-L1 elements that produced 3′ transductions in our data sets (Figs. 3, 4) also have been reported to be active in somatic tissues or in cell culture (Moran et al. 1996; Brouha et al. 2003; Beck et al. 2010; Macfarlane et al. 2013; Tubio et al. 2014). In some cases, FL-L1 elements were active in germline tissues, somatic cancers, and cultured cells (e.g., the Chr 2: 156527848 element [*LRE3*]) (Fig. 4C). However, other FL-L1 source elements behaved very differently in these three cellular contexts. For example, the Chr 22: 29059271 element was very active in somatic cancers but had very low levels of activity in germline tissues and cultured cells (Fig. 4C; Supplemental Table S9). The rates at which 5′ inversions were generated also varied in these diverse settings (Fig. 5D–F; Supplemental Table S10).

There are several factors that might help to explain these differences in FL-L1 behavior. First, FL-L1 elements occasionally can have two or more alleles that occupy the same locus, and these alleles can support very different levels of retrotransposition due to internal mutations that affect functionally important sites within L1 (Seleme et al. 2006). Under this scenario, the same FL-L1 locus might appear to have different levels of activity because different alleles of the element are being examined. A source element also could be heterozygous or homozygous at a particular locus, which might also influence the apparent rate of offspring production. In other cases, the methylation status or chromatin state of the FL-L1 element may influence the rate of retrotransposition in various tissues (Borc'his and Bestor 2004; Iskow et al. 2010; Scott et al. 2016). A range of additional host factors that are exclusively expressed in germline or somatic tissues also might be envisioned to differentially influence the activity of a given FL-L1 element. Irrespective of the mechanism(s) underlying these differences, our data suggest that FL-L1 elements likely help to shape human traits and diseases in complex ways, depending on the populations and tissues in which they are active.

## Methods

### Description of the MELT pipeline

MELT is coded in Java (release 1.8) and uses several external libraries (Supplemental Table S12). For each genome analyzed, MELT parses WGS data that is aligned with BWA-MEM or -ALN (Li and Durbin 2010) for DRPs (defined as mates that are either aligned to different chromosomes or separated by at least 1 Mbp). DRPs are then aligned to mobile element (ME) reference sequences (Supplemental Table S13; Dombroski et al. 1993; Stewart et al. 2011) using Bowtie 2 (Langmead and Salzberg 2012). DRPs where one mate maps to the human reference sequence and the other maps to a ME reference sequence (Supplemental Table S13) are then used for MEI discovery by ‘walking’ across the reference genome using the reference-aligned mate, seeking clusters of at least four DRPs. Sites are filtered based on proximity to reference MEs (Smit and Green 1996–2010; Karolchik et al. 2004), surrounding sequencing depth, location in relation to reference sequence gaps, and the mapping quality of the reads. After MEI sites are identified, DRP and SR evidence is used to discover MEI-associated features and precise breakpoints (Supplemental Table S1). MEI sites are then genotyped in all samples using a modified version of the algorithm described in Li (2011). Following genotyping, sites are filtered based on 5′ and 3′ supporting evidence, total percentage of no-call (i.e., ‘./.’) genotypes, and total number of SRs. Sites are then merged into a VCF 4.2 format file (Danecek et al. 2011).

### Simulated data sets and validation of MELT features

To facilitate in silico analyses, we generated 50 simulated human genomes with computationally inserted MEIs containing diverse features. For simulated insertions, the NA12878 genome was selected to represent a typical distribution of elements (*Alu* 922, L1 124, SVA 46). For each MEI type, a full-length consensus sequence (Supplemental Table S13; Dombroski et al. 1993; Stewart et al. 2011) was randomly modified with known ME features (Supplemental Table S1; Supplemental Fig. S2; Supplemental Methods). Using bedtools (Quinlan 2014), each MEI then was randomly inserted into an accessible locus of the hg19 genome, as demarcated by the 1000 Genomes Pilot Accessibility Mask (The 1000 Genomes Project Consortium 2015). Simulated reads were then generated with wgsim (Li and Durbin 2010) at 60× coverage (read length 100 bp, fragment length 500 bp, zero base error rate), and aligned with BWA (Li and Durbin 2009). Each BAM file was additionally down-sampled to 30×, 15×, and 7.5× with Picard Tools (http://broadinstitute.github.io/picard/) to evaluate MEI discovery performance at various coverage levels. For deletion analysis, we simulated 25 genomes with 400 *Alu* and 50 L1 polymorphic sites randomly selected from a collection of 1719 *Alu* and 139 L1 reference sites that are known to be polymorphic in humans (Smit and Green 1996–2010; Karolchik et al. 2004; Sudmant et al. 2015). We performed MEI discovery using MELT-Single (*n* = 50) and MELT-DEL (*n* = 25) (Supplemental Fig. S1). The wgsim read simulator that we used enabled us to simulate diploid human genomes, which allowed us to model both heterozygous and homozygous MEI sites. The ratio of heterozygous to homozygous non-REF MEIs was weighted according to the relative frequencies of these events in actual data (95% heterozygous and 5% homozygous non-REF). Although wgsim does not model the error profile of Illumina sequencing, the FDRs of our simulations were in good agreement with those obtained in our PCR validations, suggesting that sequencing errors likely do not have a major impact on MEI discovery (Fig. 1; Supplemental Fig. S7).

### Comparison of runtime, scalability, sensitivity, and specificity

To test the relative runtime of MELT and four additional MEI detection pipelines (Keane et al. 2013; Thung et al. 2014; Wu et al. 2014; Zhuang et al. 2014), we analyzed the NA12878 genome at ∼6× (100-bp paired-end Illumina WGS) and ∼30× (250-bp paired-end Illumina WGS) coverages without multithreading or distributed computing. Genomes were downloaded as raw FASTQ files (The 1000 Genomes Project Consortium 2015), and alignments were performed using BWA-MEM (Li and Durbin 2010). For Tangram and Mobster, Mosaik (Lee et al. 2014) alignments were generated with identical input FASTQ files and used for MEI discovery. Scalability was evaluated using the default parameters for each algorithm on one to 10 genomes with five replicates (Supplemental Table S4). Samples were added in the same order for each algorithm and each replicate. Multithreading or parallelization was enabled for algorithms that support these approaches (i.e., MELT, TEMP, and Tangram). Tangram was run with several different multithreading parameters (with either 1, 2, or 4 cores per chromosome during the tangram_detect stage) (Fig. 1; Supplemental Table S5). All reported times are actual runtimes (i.e., start to finish, not CPU time) (Fig. 1B,C; Supplemental Table S5). System specifications for the machines that were used for testing are reported in the Supplemental Methods. Sensitivity and specificity was tested in the five MEI detection algorithms using the 50 simulated data sets described above. Each tool was run according to the algorithm documentation using default parameters at four coverage levels (7.5×, 15×, 30×, and 60×) for the three human MEs (*Alu*, L1, and SVA). A site was considered correct if it fell within ±500 bp of the actual site (Quinlan 2014).

### MEI data sets and analysis

MEI discovery was performed in the 1000 Genomes Project Phase III data sets, chimpanzees, and ancient humans with MELT ver. 2.0 as outlined in the Supplemental Methods. PCR validations with 90 new MEI sites from the MELT ver. 2.0 1000 Genomes Project data set were conducted as outlined previously (Supplemental Methods; Supplemental Fig. S7; Supplemental Table S7; Sudmant et al. 2015). *Alu* subfamily annotation and stratification analysis was conducted as outlined previously (Supplemental Methods; Supplemental Table S8; Batzer et al. 1996). Analysis of L1 3′ transductions, L1 5′ inversions, L1 germline vs. somatic activity, and archaic MEIs was performed as outlined in the Supplemental Methods, Supplemental Figure S11, and Supplemental Tables S9–S11.

### Software availability

The MELT ver. 2.0 package is available at the MELT download site (http://melt.igs.umaryland.edu).

## Data access

All MELT ver. 2.0 call sets from this study have been submitted to the dbVar database at NCBI (https://www.ncbi.nlm.nih.gov/dbvar) under accession number nstd144.

## Supplementary Material

Supplemental Material
