# Supplementary material for: The Mobile Element Locator Tool (MELT): population-scale mobile element discovery and biology
Source: Genome Res. 2017 Nov;27(11):1916–29. doi: 10.1101/gr.218032.116 (PMC5668948; doi:10.1101/gr.218032.116)
Supplement: Supplemental Material [file supp_27_11_1916__index.html]

The Mobile Element Locator Tool (MELT): population-scale mobile element discovery and biology — Supplemental Material 

# The Mobile Element Locator Tool (MELT): population-scale mobile element discovery and biology

## Supplemental Material

undefined

- Supplemental\_Table\_S1.xlsx
- Supplemental\_Table\_S2.xlsx
- Supplemental\_Table\_S3.xlsx
- Supplemental\_Table\_S4.xlsx
- Supplemental\_Table\_S5.xlsx
- Supplemental\_Table\_S6.xlsx
- Supplemental\_Table\_S7.xlsx
- Supplemental\_Table\_S8.xlsx
- Supplemental\_Table\_S9.xlsx
- Supplemental\_Table\_S10.xlsx
- Supplemental\_Table\_S11.xlsx
- Supplemental\_Table\_S12.xlsx
- Supplemental\_Table\_S13.xlsx
- Supplemental\_Materials\_FINAL\_SUBMISSION.pdf
