# Supplementary material for: The Mobile Element Locator Tool (MELT): population-scale mobile element discovery and biology
Source: Genome Res. 2017 Nov;27(11):1916–29. doi: 10.1101/gr.218032.116 (PMC5668948; doi:10.1101/gr.218032.116)
Supplement: Supplemental Material [file supp_gr.218032.116_Supplemental_Material_.pdf]

Supplemental Materials

The Mobile Element Locator Tool (MELT): Population-scale mobile element discovery and biology.

Eugene J. Gardner, Vincent K. Lam, Daniel N. Harris, Nelson T. Chuang, Emma C. Scott, W. Stephen Pittard, Ryan E. Mills, 1000 Genomes Project Consortium, and Scott E. Devine.

Table of Contents

1.	**Supplemental Methods**	**Page**
	Testing the performance of MELT in new compute environments	2
	Generation of simulated data sets and validation of MELT features	2
	Quality tranche system	2
	Sensitivity, specificity, and runtime of MELT and other algorithms	3
	Generation of MELT MEI call sets	3
	PCR validation of MELT	3
	Identification of population-specific *Alu* subfamilies	4
	Assessment of *Alu* allelic heterogeneity	4
	Pacific Biosciences sequencing of FL-L1 elements	5
	L1 3' transduction tracking	5
	Detection of 5' inversions	5
	Archaic hominid population genetics	5
2.	**Supplemental Figures**	
	Supplemental Figure S1	7
	Supplemental Figure S2	8
	Supplemental Figure S3	9
	Supplemental Figure S4	10
	Supplemental Figure S5	11
	Supplemental Figure S6	12
	Supplemental Figure S7	13
	Supplemental Figure S8	14
	Supplemental Figure S9	15
	Supplemental Figure S10	16
	Supplemental Figure S11	17
	Supplemental Figure S12	18
3.	**Supplemental Results and Discussion**	
	Interior mutation rates in MEIs	19
	Analysis of alternative poly(A) signals for 3'-transducing FL-L1 elements	19
	FDRs in MELT 1.0 vs. 2.0	19
	Germline vs. somatic MEIs	20
	MEIs as tools for population genetics	20
	Chimpanzee MEI call sets	20
4.	**Supplemental References**	21

Supplemental Methods

Testing the performance of MELT in new compute environments

We developed benchmarking tests to ensure that the different implementations of MELT ver. 2.0 are comparable and stable in diverse compute environments. A standardized test for this purpose is to run MELT on the 6X NA12878 genome and evaluate the MEIs and associated features that are called. For example, we compared the Amazon AMI cloud version of MELT-SGE with the local version of MELT-SGE using NA12878 as the test genome and found comparable results: 4001/4007 (99.8%) of the MEIs and associated features were called identically by these two versions of MELT. The clock speeds also were comparable (10.7 vs. 11.0 minutes). For each new implementation of MELT, we have conducted similar tests to ensure uniform performance. We have installed and used MELT 2.0 at all three of the authors' institutions (University of Maryland School of Medicine, University of Michigan Medical School, and Emory University) and have received user feedback from a range of institutions reporting similar findings.

Generation of simulated data sets and validation of MELT features (See also Methods)

Inserted mobile elements were modified with the following features (Supplemental Table S1, Supplemental Fig. S2): poly(A) tail length (*Alu* 0 – 75 bp, SVA 0 – 50 bp, L1 0 – 50 bp), MEI length (*Alu* 281 bp, SVA 400 – 1627 bp, L1 length 100 - 6019), strand (positive or negative in equal representation), and target site type (duplication 95%, deletion 2.5%, zero length 2.5%). For *Alu* insertions, the consensus sequence was modified to match one of ten different *Alu* subfamilies. Additionally, 5% of L1 insertions were reverse-complemented for a random length starting at the 5' end to simulate 5' inversions (Ostertag and Kazazian 2001). Genotypes were randomly selected, with 95% inserted as heterozygous and 5% inserted as homozygous. For all MEI types, length, orientation, breakpoint precision, and genotyping were assessed. The accuracy of subfamily assignment was evaluated for *Alu* elements and the accuracy of twin-priming detection was evaluated for L1 elements. Simulations of 3' transductions (Moran et al. 1999) were carried out by first inserting 100 L1s with associated 3' transductions randomly into the reference human genome and then testing MELT's ability to detect these MEIs and the 3' transductions at 7.5X, 15X, 30X, and 60X simulated coverages. Ten independent replicates of each test were performed. These data are plotted in Supplemental Fig. S4F. Simulation tests to determine whether MELT had sufficient sensitivity to detect 5' inversions within the first 590 bp of L1 were conducted by generating 100 L1s with 5' inversions in the first 590 bp of L1 and inserting these randomly into the reference human genome. MELT's ability to detect these MEIs and associated 5' inversions was tested at 7.5X, 15X, 30X, and 60X simulated coverages. Ten independent replicates of each test were performed. At simulated sequence coverages of 7.5X, we detected 659/1000 (65.9%) of the test elements that carried 5' inversions in the first 590 bp of L1 (Supplemental Fig. S4H; Supplemental Table S6). Therefore, given that the average sequence coverage for the 2,504 genomes that were sequenced by the 1000 Genomes Project is very similar (7.4X; Sudmant et al. 2015), and that we discovered 1,440/4,118 L1's that contained the first 590 bp of L1 in the 1000 Genomes samples, we would expect to discover ~1440 X 0.659 = 949 inversions within the first 590 bp of L1. However, we actually detected zero 5' inversions in this region. These simulations demonstrate that we could indeed detect 5' inversions in the first 590 bp of L1 if they existed, and that there is a true underrepresentation of 5' inversions in this region.

Quality tranche system

We developed a quality tranche system that provides a score to each MEI call on the basis of the amount of evidence that was used to detect the MEI. The quality scores range from 5 (best) to 0 (worst) and are provided in the VCF file to allow the user to estimate the relative quality of a given MEI breakpoint (Supplemental Table S2). Tranche score is directly related to the level of sequence coverage per sample. At 60X coverage, most calls fall within the highest quality tranche (5), whereas at lower coverages, the number of calls in the lower tranches (0-4) increases (Supplemental Fig. S4).

Population sizes where a large amount of evidence is available for MEI sites similarly have an increased number of calls in the highest quality tranche 5.

Sensitivity, specificity, and runtime of MELT and other algorithms

MEI discovery on the 6X coverage genome and the 30X coverage genome was performed a total of five times at each coverage using default parameters for each algorithm on a Dell Precision T3600 desktop running Red Hat Enterprise Linux release 5.11 with a four core Intel Xeon 3.6Ghz processor and 16Gb DDR3 RAM with no network connectivity (Figure 1A; Supplemental Table S5). Mobster was unable to complete MEI discovery for the 30X coverage genome due to an out of memory error, even when 14Gb of total memory was provided to the java virtual machine (*-Xmx 14G*). Thus, Mobster is listed in Figure 1A as did not finish (DNF).

Population-scale analyses were performed using a cluster consisting of two Dell PowerEdge M620 blades with identical configurations (2 Intel Xeon E5-2695 CPUs with 12 dual-threaded cores and 128 Gb of DDR3 RAM). Due to high variability in total runtime for TEMP, the two longest runs were excluded in Figure 1B (all times are listed in Supplemental Table S5, with excluded times marked in red). All algorithms except for RetroSeq were examined in the population scaling tests, as RetroSeq was not designed for population scale analysis.

Generation of MELT MEI call sets

To generate MEI call sets, we first downloaded the following data from public data repositories: 2,534 1000 Genomes Project BAM files (1000 Genomes Project Consortium 2015), 25 Great Ape Project chimpanzee BAM files (Prado-Martinez et al. 2013), one Neanderthal BAM file (Prufer et al. 2014), and one Denisovan BAM file (Meyer et al. 2012; Supplemental Table S4). Data from chimpanzees and ancient individuals were converted to FASTQ format and then realigned to the panTro4 or hg19 reference, respectively, using BWA-MEM version 0.7.9a-r786, with default settings (Li and Durbin 2009). Biostar84452 (available from: http://lindenb.github.io/jvarkit/) was used to trim split reads, such that the reads were no longer split, in the Neanderthal and Denisovan genome alignments. Duplicates were then marked in all alignments with the MarkDuplicates tool found within the Picard Tools package (http://broadinstitute.github.io/picard/). Prior to MELT analysis, several human and chimpanzee samples were filtered due to an excessive number of DRPs (Supplemental Table S4). MELT discovery was performed using MELT-SGE (Supplemental Fig. S1) with default parameters in all cases, except for archaic discovery, where –cov was set to 5. Only PASS sites were included in final VCF files. MEIs that could not be genotyped (. / .) in both archaic individuals were filtered for all downstream archaic analyses. Human and chimpanzee BAM files were additionally ascertained for presence or absence of reference *Alu* or L1 MEIs using MELT-DEL (Supplemental Fig. S1). The new 1000 Genomes Project MELT MEI calls that were generated with MELT ver. 2.0 were compared to the MEI calls that were originally generated for Phase III of the 1000 Genomes Project with MELT ver. 1.0 (1000 Genomes Project Consortium 2015; Sudmant et al. 2015) using a +/- 500 bp overlap (Supplemental Fig. S6; Quinlan 2014). Chimpanzee MEI calls were compared to previously published chimpanzee MEI data using the same approach (Supplemental Fig. S8; Hormozdiari et al. 2013).

PCR validation of MELT

Although MELT ver. 1.0 was validated extensively with PCR assays as part of the 1000 Genomes Project (Sudmant et al. 2015), we wanted to ensure that the improvements that we introduced into MELT ver 2.0 did not significantly alter the accuracy of MEI discovery. The simulation studies that we performed with MELT ver. 2.0 indicated good performances in terms of sensitivity and specificity (Figure 1). We also performed PCR validation of 90 MEI sites (31 *Alu*, 31 L1, and 28 SVA; Supplemental Table S7) that were discovered with MELT ver. 2.0. Sites and primers are reported in Supplemental Table S7. PCR amplification was performed using Qiagen Taq DNA Polymerase (Qiagen catalog #: 201203). All experiments included i) a genomic DNA sample (gDNA) that was expected to have the MEI based on the MELT calls, ii) a gDNA sample that was expected to lack the

insertion based on the MELT calls, and iii) one PCR reaction that lacked gDNA. PCR reaction conditions were as follows: 3m at 94°C followed by 32 cycles of 30s at 94°C, 30s at 57°C, and 1m at 72°C with a final elongation for 10m at 72°C. A test was considered positive if a PCR product of the expected size was observed only in the individual that was predicted by MELT to have the insertion (Supplemental Fig. S7; Supplemental Table S7). After initial testing, L1 sites that were negative were assessed again using an 'A' + 'D' long-range PCR approach to rule out the possibility of internal sequence changes preventing the binding of the 'C' primer. Long-range PCR using LA Taq DNA Polymerase (Clonetec catalog #: RR002M) was performed for each L1 with the following reaction conditions: 1m30s at 94°C followed by 32 cycles of 30s at 94°C, 30s at 57°C, and 8m30s at 68°C with a final elongation for 10m at 68°C. Sites where this approach was used are listed in Supplemental Table S7. Please also see Supplemental Results and Discussion (below).

Identification of population-specific *Alu* subfamilies

For each *Alu* element that was discovered in this study, the internal sequence was assembled as part of standard MELT analysis. The assembly was analyzed using the C*Alu* algorithm, which is included in MELT ver. 2.0, and the fully-assembled sequences are provided in the MELT VCF file in the MEINFO and DIFF fields in the VCF INFO column. C*Alu* classifies *Alu* elements according to subfamily using interior SNPs (Roy et al. 2000; Bennett et al. 2008; Konkel et al. 2015). When an *Alu* cannot be assigned to a known subfamily (e.g. *Alu*Ya5, *Alu*Yb8, etc.) due to ambiguity in the assembled sequence, C*Alu* instead assigns elements to ancestral families (e.g. *Alu*Ya, *Alu*Yb, or *Alu*Y), if possible. All *Alu* sites discovered in this study were annotated and displayed in Krona plots (Supplemental Fig. S9; Ondov et al. 2011).

Additional single nucleotide variants, beyond those that define known families and subfamilies (Roy et al. 2000; Bennett et al. 2008; Konkel et al. 2015), also were discovered in *Alu* elements. To prevent biases that may be caused by gaps in assembled interior sequences, only *Alu* sites with at least 90% of the interior sequence assembled at greater than 2X coverage were included in this analysis (10,003/17,543; 57.0%). *Alu* elements with identical internal sequences (including specific bp changes) were grouped to identify new subfamilies. To control for random expansion and contraction of the A-rich linker in the *Alu* consensus sequence, insertions at position 127 were excluded from this analysis. Additionally, families defined by only a single CpG change with fewer than 20 members were excluded from this analysis. Groups with at least five members then were analyzed for total allele count across all 1000 Genomes Project continental populations (Figure 2; Supplemental Table S8). To determine allelic sharing within these new families, each site within a family was assessed for total allele count in the four major non-admixed continental populations in the 1000 Genomes Project (AFR, SAS, EAS, EUR).

To determine groups with exceptional differences in frequencies among continental populations, we calculated χ^2 values for all groups using GraphPad Prism (version 6.0g for Mac OSX, GraphPad Software, San Diego California USA, www.graphpad.com). χ^2 values were calculated as a contingency table with "observed" represented as allele count in each continental population and "expected" represented as a sum of all four populations divided by four (Supplemental Table S8).

Assessment of *Alu* allelic heterogeneity

To determine whether individual *Alu* loci accumulated differences in their internal sequences after they were inserted into the human genome, we ran MELT on five high coverage genomes (Supplemental Table S4) and assessed the supporting reads for each *Alu* site with an allele count that was greater than one. Using these data, we genotyped each non-CpG base in the reference *Alu* sequence across all individuals in which an *Alu* insertion was present. Bases where we could not identify a single majority genotype at each position then were summed and are reported in Supplemental Table S8. Using these data, we then calculated a per base mutation rate using the following formula:

$$\frac{Total\ bases\ mutated\ across\ all\ Alu}{Total\ bases\ assessed * Total\ allele\ count}$$

Pacific Biosciences sequencing of L1 elements

L1s were PCR-amplified, sequenced using the Pacific Biosciences RS II, and then assembled using the method described in Scott et al. (2016). Assembled sequences were compared to other offspring from the same source element and are reported in Figure 3F and Supplemental Table S9.

L1 3’ transduction tracking

Source-offspring relationships were tracked for both reference and non-reference FL-L1 elements using the ‘Transduction’ tool that is found in the MELT ver 2.0 package. This algorithm searches the 3’ ends of both REF (Karolchik et al. 2004) and non-REF FL-L1s (defined as any L1 greater than 5,900bp in length) for DRPs that map to another locus in the human genome immediately adjacent to a known non-reference L1 (Supplemental Table S9). These relationships are reflected in the VCF output in the ‘SOURCE’ flag of the ‘INFO’ column. We validated 18 offspring that contained 3’ transductions using our Pacbio sequencing approach (Scott et al. 2016). 17/18 (94.4%) were validated with this approach, and the complete sequences of these elements, including the 3’ transductions, are included in Supplemental Table S9. Each FL-L1 element that gave rise to a 3’ transduction was manually analyzed using raw sequencing data to identify a canonical poly(A) signal (either AAUAAA or AUUAAA; Zhao et al. 1999) when possible, and a new downstream poly(A) signal at the putative site of 3’ transduction (Supplemental Table S9).

To compare the FL-L1 source elements that were identified in this study with those that were previously published, we conducted a comprehensive literature survey and compiled a table of these elements (Supplemental Table S9; Dombroski et al. 1991; Holmes et al. 1994; Brouha et al. 2002; Myers et al. 2002; Beck et al. 2010; Kidd et al. 2010; Evrony et al. 2012; Solyom et al. 2012a; Solyom et al. 2012b; Macfarlane et al. 2013; Helman et al. 2014; Pitkanen et al. 2014; Tubio et al. 2014; Scott et al. 2016). Only sites that gave rise to at least one offspring insertion were included in this analysis, as determined by either a 5’ or 3’ transduction, or through the use of interior mutations (Scott et al. 2016). Orphan sites were not included. Source elements described in Kidd et al. (2010) were obtained from the Eichler lab. Source elements were classified as active in the germ-line, somatic cells, or both as outlined in the reporting publication(s) and this study. Activities of elements tested in cell culture were obtained from Brouha et al. 2002, Brouha et al. 2003, or Beck et al. 2010 and are noted in Supplemental Table S9. Please also see Supplemental Results and Discussion below.

Detection of 5’ inversions

To identify 5’ inversions in L1 elements, we extracted all sites where the ‘ISTP’ INFO flag in the MELT VCF was greater than 0. Sites were then placed in histogram bins according to the site of 5’ inversion in relation to the LINE-1 reference sequence, and compared to the overall LINE-1 length distribution ascertained by MELT (Figures 5A-C). To compare our calculated rates of 5’ inversion among different tissue types, we surveyed published literature on germline and somatic L1 activity (Supplemental Table S10). Papers that did not measure the 5’ inversion status of L1’s were excluded from analysis.

Archaic hominid population genetics

Using the archaic MEI VCF generated by MELT, we sought to determine allelic sharing of *Alu* MEIs between modern humans, archaic individuals, and chimpanzees (Figure 6A, B; Supplemental Table S11). Following quantification of shared sites at the class level, we then determined sharing for each modern human individual using the equation:

$$\%\ Sharing\ Individual = \left(\frac{Number\ of\ MEIs\ Shared\ With\ Ancients}{Individual's\ Total\ Number\ of\ MEIs}\right) * 100$$

Percent sharing for a given MEI in a population was calculated as the mean of sharing for all individuals in that population. A two-way ANOVA with multiple-comparisons was used to determine if the mean percent sharing was significantly different between populations using GraphPad Prism, version 6.0g. Reported Tukey-corrected multiple-comparison p-values are considered significant at $p \leq 0.05$ (Figure 6C, D; Supplemental Table S11). Sharing of MEIs between modern humans and Neanderthal was evaluated using Neanderthal haplotypes determined for all individuals studied in the 1000 Genomes Phase I project (Sankararman et al. 2014). All *Alu* (n= 42) and L1 (n = 7) MEIs that were shared with Neanderthals and not present in African individuals were intersected with Neanderthal haplotypes using the bedtools intersect function (Quinlan 2014). MEIs were then classified into one of several categories based on the combination of an MEI and Neanderthal haplotype. r^2 analysis was carried out between the Neanderthal haplotype and the MEI as described previously (Rogers and Huff 2009). To serve as a control, we repeated the above analysis using *Alu* (n = 53) and L1 (n = 7) MEIs that were shared between Neanderthals and modern humans, with an African allele frequency greater than zero (Figure 6F; Supplemental Table S11).

Supplemental Figures

Supplemental Fig. S1

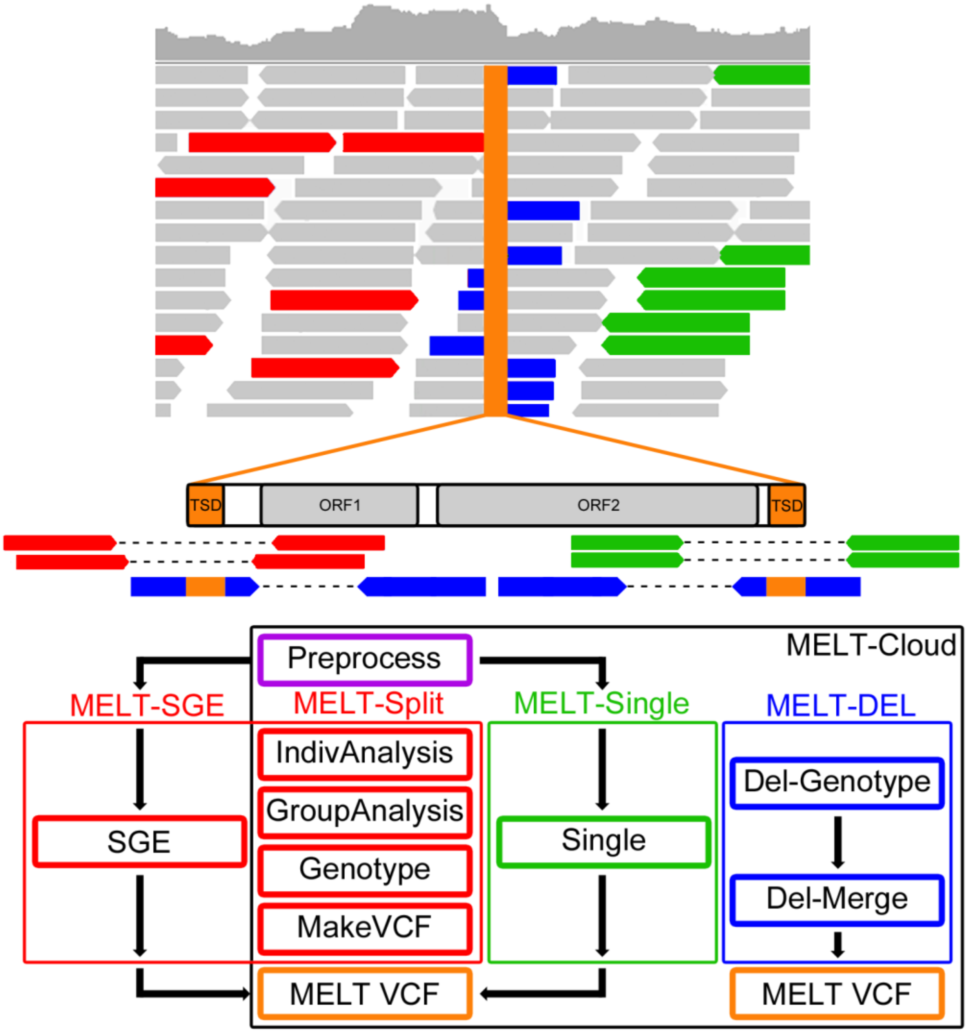

Supplemental Fig. S1. MELT pipeline overview.

MELT performs MEI discovery using Illumina WGS paired end reads. (**A**) MELT uses two types of evidence to ascertain the location of MEIs: discordant read pairs (DRPs) and split reads (SRs). MELT first uses DRPs that map to both the reference genome (top panel) and an ME sequence (bottom) on both the left (red arrows) and right (green arrows) side of the insertion site to determine the approximate location of an MEI. MELT then uses SRs (blue arrows) that align to both the reference genome (top) and the ME (bottom panel) to determine the precise location of the insertion site and the target site duplication (TSD; Orange). (**B**) MELT performs non-reference and reference MEI discovery through multiple processing pipelines. Analysis of population scale data (red box) can be performed using either the built-in SGE scheduler (MELT-SGE), or adapted to other parallel computing environments (using MELT-Split). MELT also can rapidly analyze a single genome (green box) using MELT-Single, or genotype reference MEIs (blue box) using the MELT-DEL pipeline. An AMI version of MELT ver. 2.0 also is available at Amazon Web Services (AWS) to facilitate cloud-

based MEI discovery.

Supplemental Fig. S2

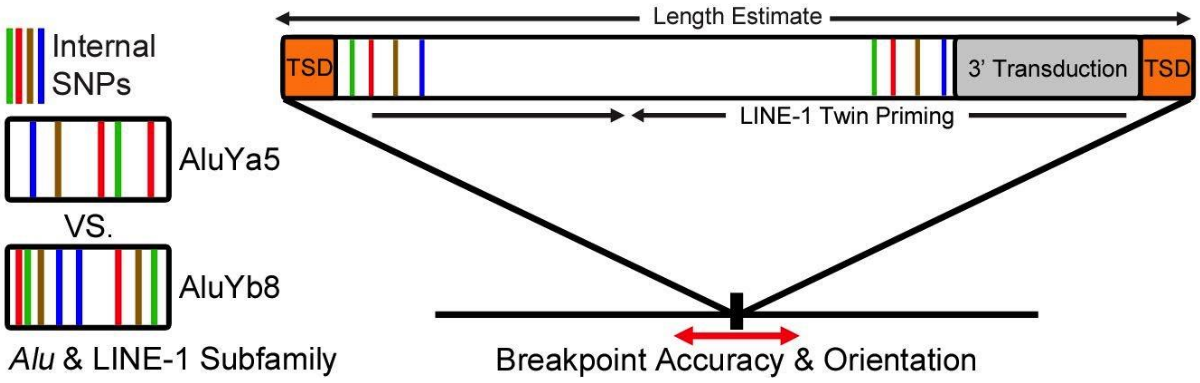

Supplemental Fig. S2. Cartoon of features described in Supplemental Table S1. Shown is a typical L1 insertion with flanking target site duplications, a 3' transduction, and interior sequence changes (green, red, brown, blue colored bars). Shown at left are *Alu* elements with different interior changes (colored bars) that are classified by the C*Alu* algorithm. A similar tool, called LINEu, evaluates interior mutations in L1 elements.

Supplemental Fig. S3.

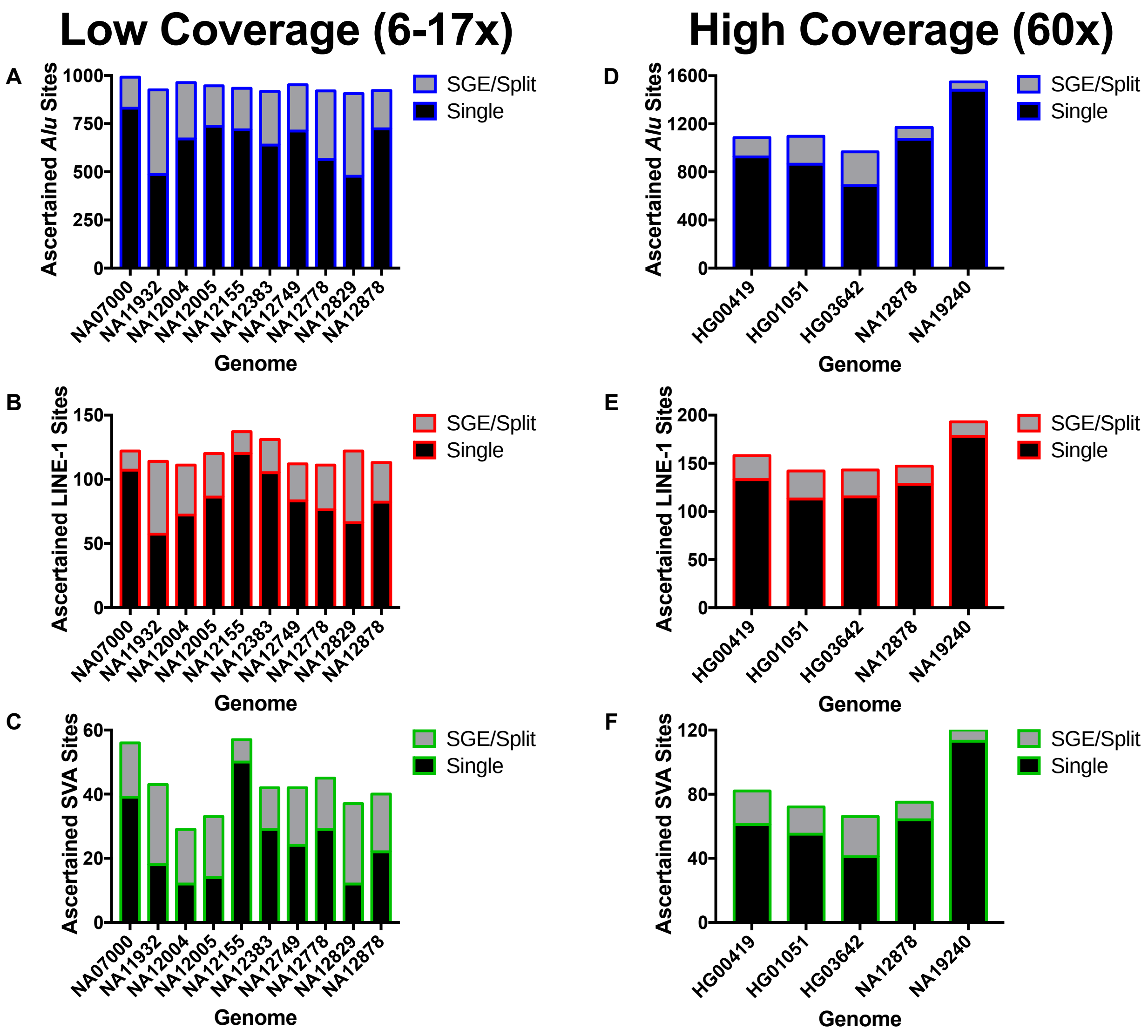

Supplemental Fig. S3. Comparison between MELT-Single and MELT-SGE.

Comparison of MELT ascertained sites for all three MEI classes (*Alu*, L1, SVA) when running genomes separately with MELT-Single versus as a batch with MELT-SGE. Note that MELT-SGE and MELT-Split achieve equivalent results and differ only in the level of automation of job scheduling. (**A-C**) Low (6-17X) coverage ascertainment of (**A**) *Alu*, (**B**) L1, and (**C**) SVA in ten CEU genomes. Black represents sites found by running MELT-Single separately on each genome (X-axis). Grey represents additional sites discovered in each genome when all genomes listed on the X-axis are analyzed together using MELT-SGE. (**D-F**) The same comparisons for (**D**) *Alu*, (**E**) L1, and (**F**) SVA in five high coverage (60X) genomes using MELT-Single vs. MELT-SGE. All of these genomes were sequenced by the 1000 Genomes Project and are listed in Supplemental Table S4.

Supplemental Fig. S4.

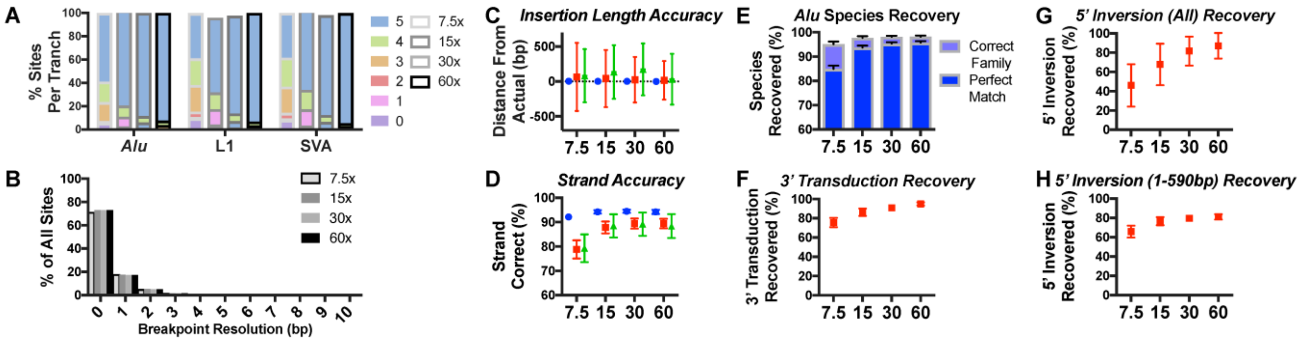

Supplemental Fig. S4. *in silico* validation of MELT MEI feature discovery. Accuracy of MELT feature assessment (Supplemental Table S1; Supplemental Fig. S2) using simulated genomes at four coverage levels (7.5X, 15X, 30X, and 60X; See Methods; Supplemental Table S6). (**A**) MELT classifies breakpoints into one of 6 tranches on a scale from 0-5, with '0' being the least accurate and '5' being the most accurate (Supplemental Table S2). At 7.5X coverage, 59.7% of *Alu* sites, 37.6% of L1 sites, and 37.4% of SVA sites, fall into tranche 5. Increased sequence coverage improves this distribution, with >90% of sites in tranche 5 at 60X coverage. (**B**) Aggregate accuracy of MELT breakpoints. At 7.5X coverage, MELT accurately reports 71.4% of MEIs to within breakpoint resolution. (**C**) Insertion length accuracy. Insertion length at 7.5X coverage is, on average, within 5, 65, and 82bps for *Alu* (blue), L1 (red), and SVA (green), respectively, improving at 60X coverage to within 1, 16, and 32bps, respectively. (**D**) Insertion strand assessment. Even at low coverage, MELT reports the correct MEI orientation for 92.2%, 78.8%, and 79.2% of *Alu*, L1, and SVA elements, respectively. Increased (60X) coverage leads to further improvements to 94.2%, 89.4%, and 88.4% for *Alu*, L1, and SVA elements, respectively. (**E**) *Alu* subfamily classification using the C*Alu* algorithm. MELT correctly assembles and assigns 84.9% of *Alu* elements to the appropriate subfamily at 7.5X coverage (e.g. *Alu*Ya5, *Alu*Yb8, etc. - Dark Blue). MELT classifies an additional 10.2% of sites at 7.5X coverage to the correct *Alu* family (e.g. *Alu*Ya, *Alu*Yb, etc. - Light Blue). At 60X coverage, 95.8% sites are assigned to the correct subfamily. (**F**) 3' Transduction recovery for L1 elements. MELT detects 75.5% of offspring elements with 3' transductions at 7.5X coverage and this improves to 94.9% at 60X coverage. (**G-H**) L1 twin priming recovery. (**G**) At low coverage, MELT recovers 44.6% of L1 elements with inverted 5' ends, and this improves to 82.2% at 60X coverage. (**H**) MELT also accurately recovers 65.9% of simulated 5' inversions in the first 590 bp of L1 at 7.5X coverage, and this improves to 81.2% at 60X coverage.

Supplemental Fig. S5.

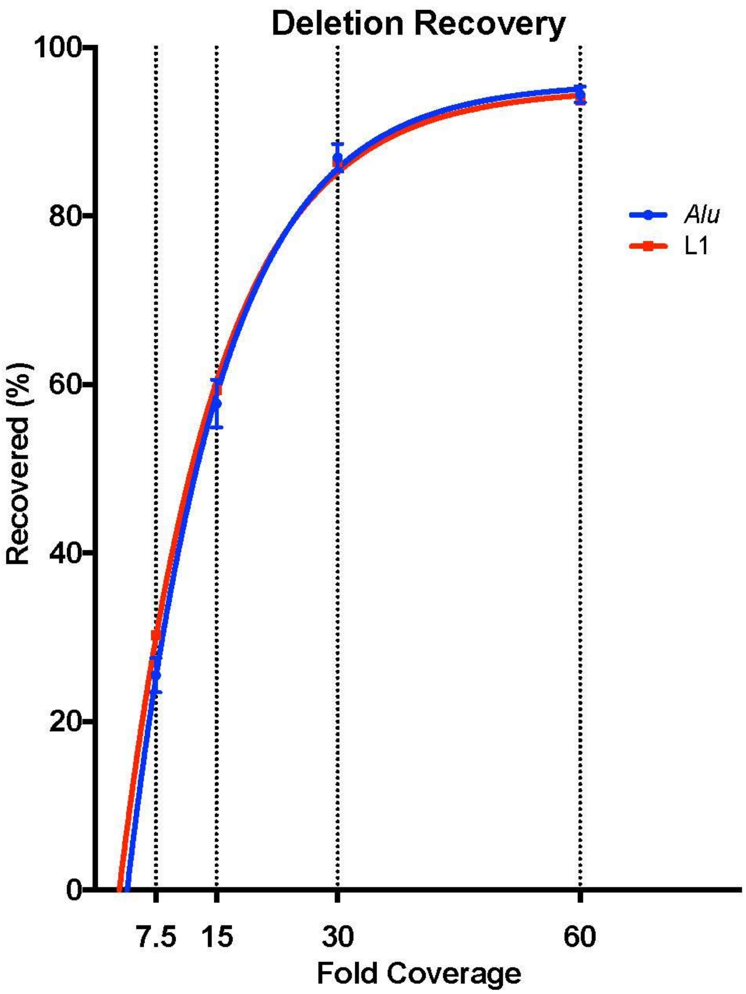

Supplemental Fig. S5. MELT-DEL accuracy. To determine the sensitivity of the MELT-DEL algorithm, we simulated 25 genomes at four different coverage levels (7.5X, 15X, 30X, 60X) with reference ME deletions (see Methods). Shown is the aggregate accuracy of MELT-DEL at all four coverage levels for *Alu* and L1 reference ME deletions (Supplemental Table S6).

Supplemental Fig. S6.

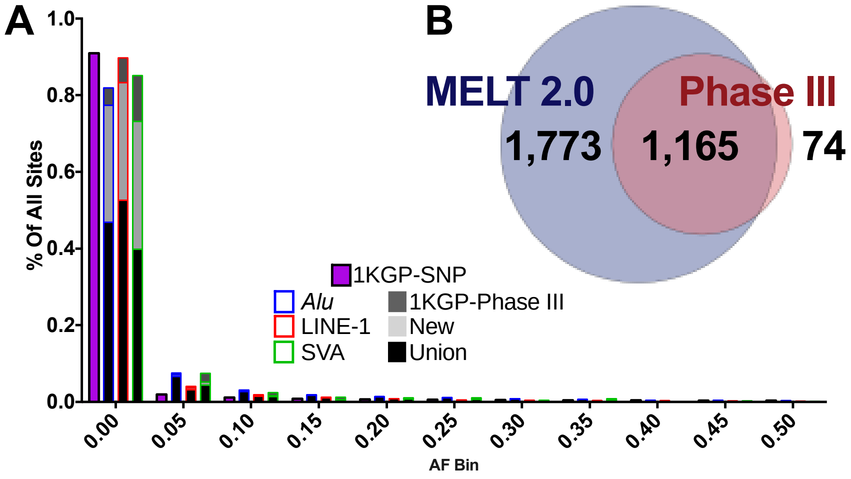

C

MELT 2.0 1KGP Phase III Discovery			
	1KGP Phase III	This Study	% Increase
Alu	12,749	17,543	37.60%
**LINE-1**	3,049	4,118	35.06%
**SVA**	835	1,062	27.03%
**Total**	16,634	22,723	36.61%

Supplemental Fig. S6. Discovery of MEIs in the 1000 Genomes Project Phase III samples. (**A**) Comparison between MELT versions 2.0 and 1.0 (Sudmant et al. 2015) for human MEIs (*Alu* - blue, L1 - red, SVA - green) in 0.05 allele frequency bins. Shown are sites discovered with both algorithms (black), MELT 2.0 only (light grey), and MELT 1.0 only (dark grey). MELT 2.0 discovered 36.6% more MEIs than MELT 1.0, while maintaining similar levels of accuracy. For comparison, we plotted the SNP allele frequencies from the 1KGP (1000 Genomes Project Consortium, 2015) in the same 0.05 allele frequency bins (purple). (**B**) MELT deletion (MELT-DEL) calls in the reference human genome and comparison between MELT-DEL and reference MEIs determined to be polymorphic by the 1KGP (Sudmant et al. 2015). MELT-DEL recovered an additional 1,773 polymorphic REF sites beyond those published by the 1KGP, while also recovering 1,165 (94.0%) of the sites that were published in that study. A significant number of these new sites (205 or 6.52%) are polymorphic reference L1s, a class that wasn't well characterized by the 1KGP. (**C**) Tabulation of the differences between MEI discovery performed by MELT 1.0 (Sudmant et al. 2015) and MELT 2.0 (this study).

Supplemental Fig. S7.

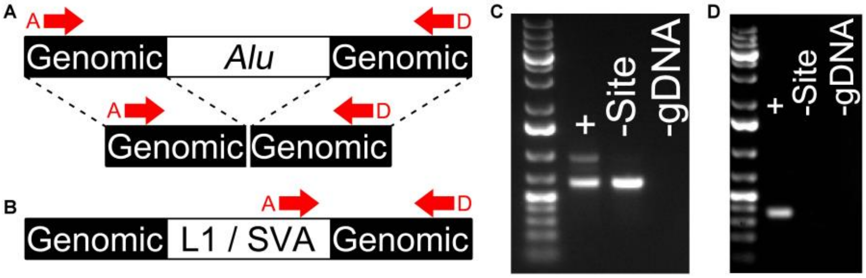

E

PCR Assessed MELT FDR			
	1KGP FDR	Adj. 1KGP FDR	New Sites FDR
Alu	2/64=**3.13**%	0/61=**0.00**%	2/31=**6.45**%
L1	2/54=**3.57**%	1/49=**2.04**%	2/31=**6.45**%
SVA	7/59=**11.86**%	6/50=**12.00**%	3/28=**10.71**%

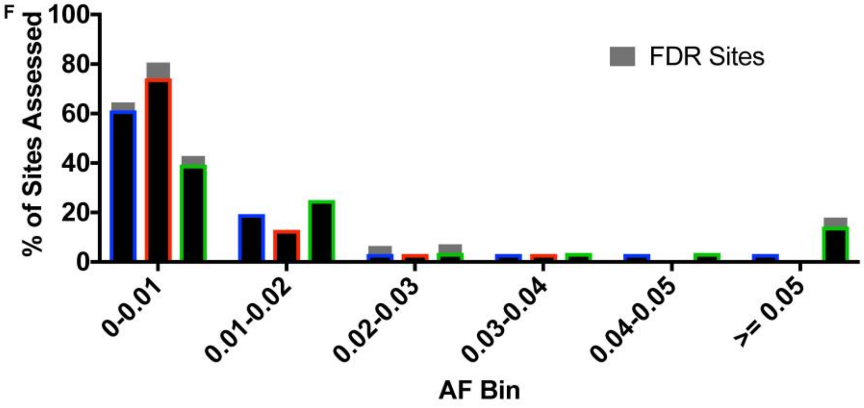

Supplemental Fig. S7. MELT PCR validation. (**A**,**B**) PCR validation diagrams. (**A**) To validate *Alu* insertions, we used two primers flanking each *Alu* site (red arrows labeled A and D; Supplemental Table S7). (**B**) To validate L1 and SVA sites, an internal primer specific to the ME class was used in combination with an external genomic primer (red arrows labeled A and D; Supplemental Table S7). (**C**,**D**) Examples of positive PCR tests. (**C**) An *Alu* test was considered positive if both an empty site and filled site (representing a heterozygous insertion), or just a filled site (representing a homozygous site) was amplified exclusively in the individual that should be positive for the insertion. Shown is an example of a positive heterozygous test. (**D**) An L1 or SVA test was considered positive if a band could be amplified of the appropriate size exclusively in the individual that should be positive for the insertion. Shown is an example of a positive L1 test. (**E**) PCR assessment of the MELT false discovery rate (FDR). As part of the 1KGP, an independent group assessed the FDR of MELT (1KGP FDR; Sudmant et al. 2015). We also have provided an adjusted 1KGP FDR based on sites were not identified by MELT ver. 2.0 (Adj. 1KGP FDR). To ensure MELT maintained similar FDRs following the improvements that were introduced into MELT ver. 2.0, we performed PCR validations on an additional 90 sites (31 *Alu*, 31 L1s, and 28 SVAs) that were discovered with MELT ver. 2.0 (Supplemental Table S7). The PCR validation rates were comparable for MELT ver. 1.0 and 2.0. (**F**) Frequency distribution bins for MELT 2.0 sites examined in our PCR validation study. Sites were placed into 0.01 allele frequency bins (X-axis). Sites that were determined to be incorrect are shown in grey above each bar.

Supplemental Fig. S8.

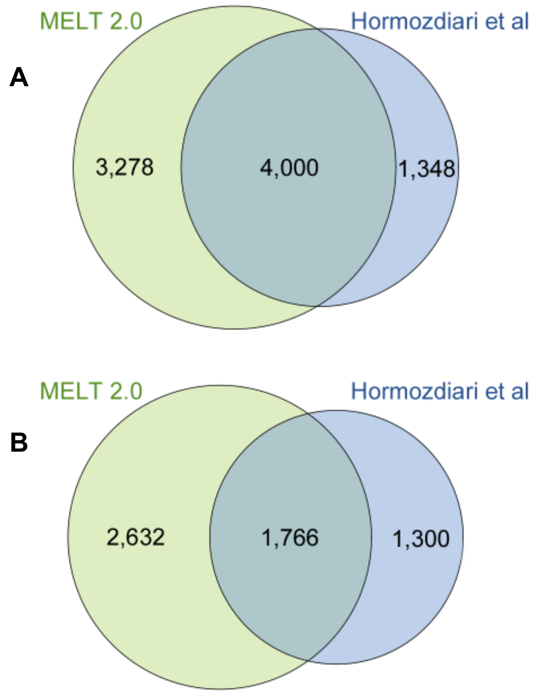

Supplemental Fig. S8. Comparison of chimpanzee MEIs discovered in this study and in Hormozdiari et al. 2013. MELT ver. 2.0 was run on 24 *Pan troglodytes* WGS samples that also were analyzed in Hormozdiari et al. (2013) to identify non-reference chimpanzee MEIs. (**A**) Comparison of *Alu* discovery. (**B**) Comparison of L1 discovery. Differences in MEI discovery between the two studies likely were caused by several factors, including: 1) differences in the REF genomes that were used to map chimpanzee traces for MEI discovery (panTro4 for MELT vs. the human genome for Hormozdiari et al.), 2) errors in liftover coordinates created during the conversion of human genome REF coordinates to panTro4 coordinates, and 3) differences in sensitivities and specificities of the MEI discovery pipelines (MELT vs. a modified version of VariationHunter).

Supplemental Fig. S9.

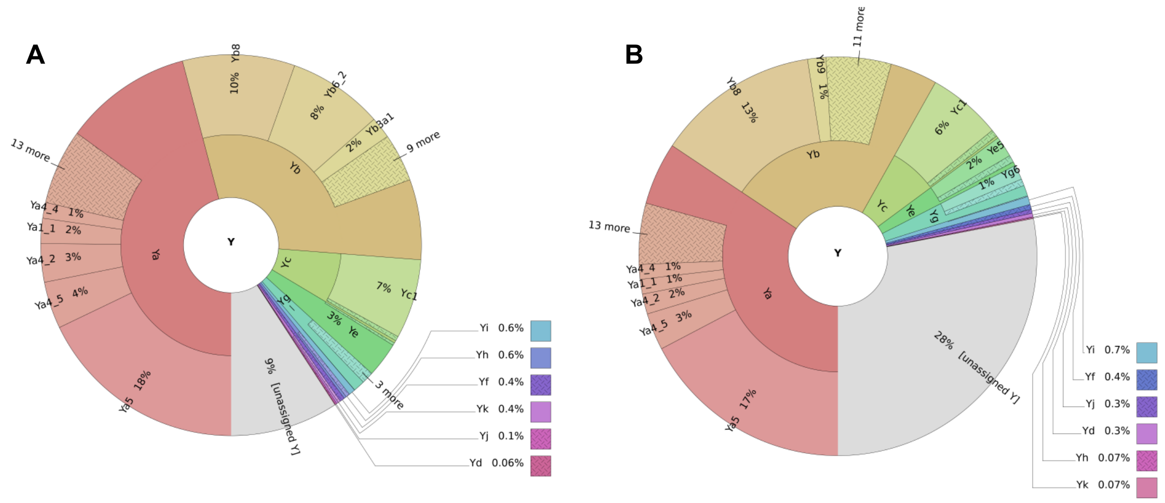

Supplemental Fig. S9. Krona plots of *Alu* family and subfamily classifications. Shown are Krona plots (Ondov et al. 2011) depicting *Alu* family classification for both (**A**) non-reference and (**B**) reference polymorphic insertions. C*Alu* will only classify at the subfamily level when the assembled sequence lacks ambiguous bases. When ambiguous bases are present, C*Alu* will place the element into a lineage rather than a subfamily (i.e. *Alu*Ya), where possible. Krona plots reflect this by allowing for multiple classification ‘tiers’, represented by the labeled concentric circles.

Supplemental Fig. S10.

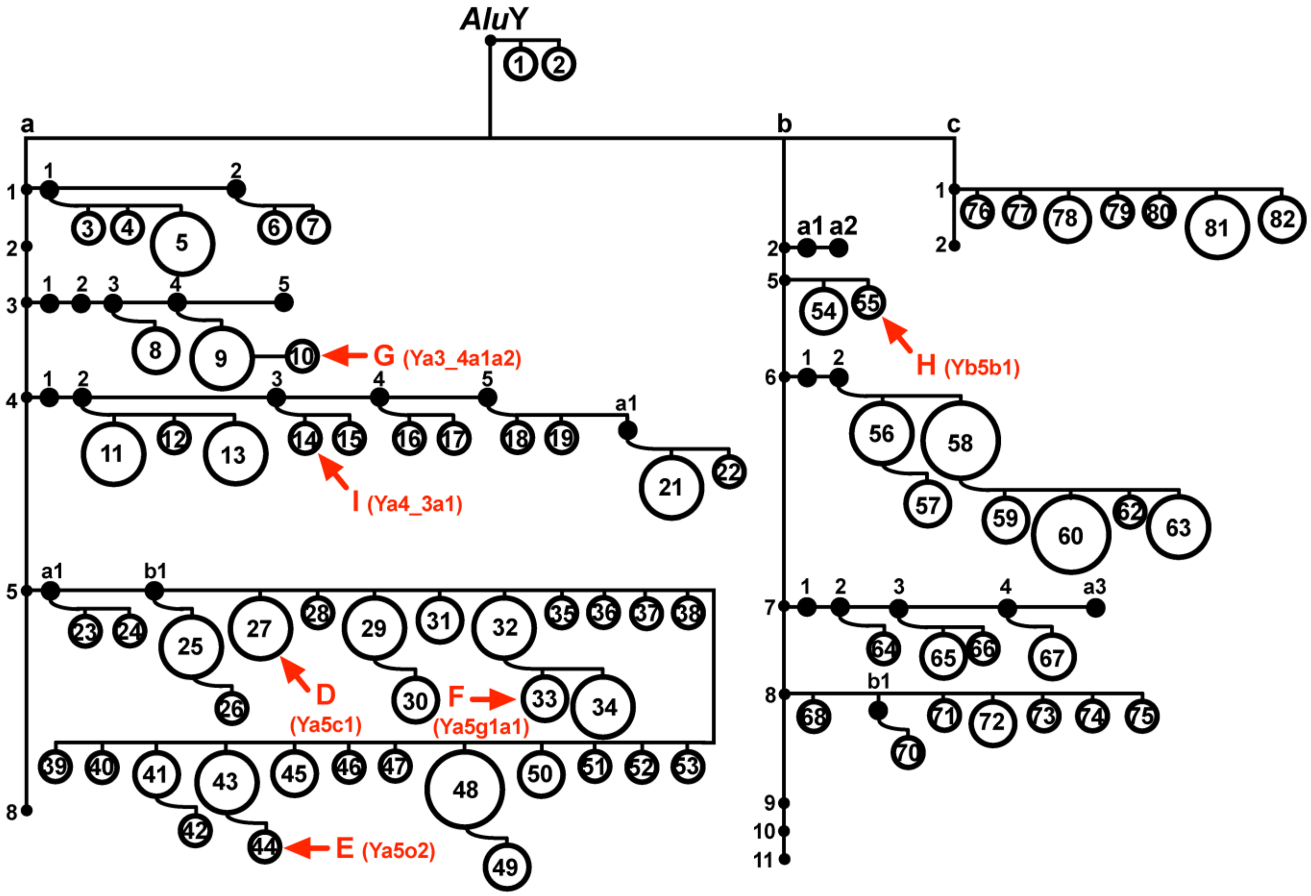

Supplemental Fig. S10. Key for Figure 2C. The numbers within each circle represent one column in Supplemental Table S8.

Supplemental Fig. S11.

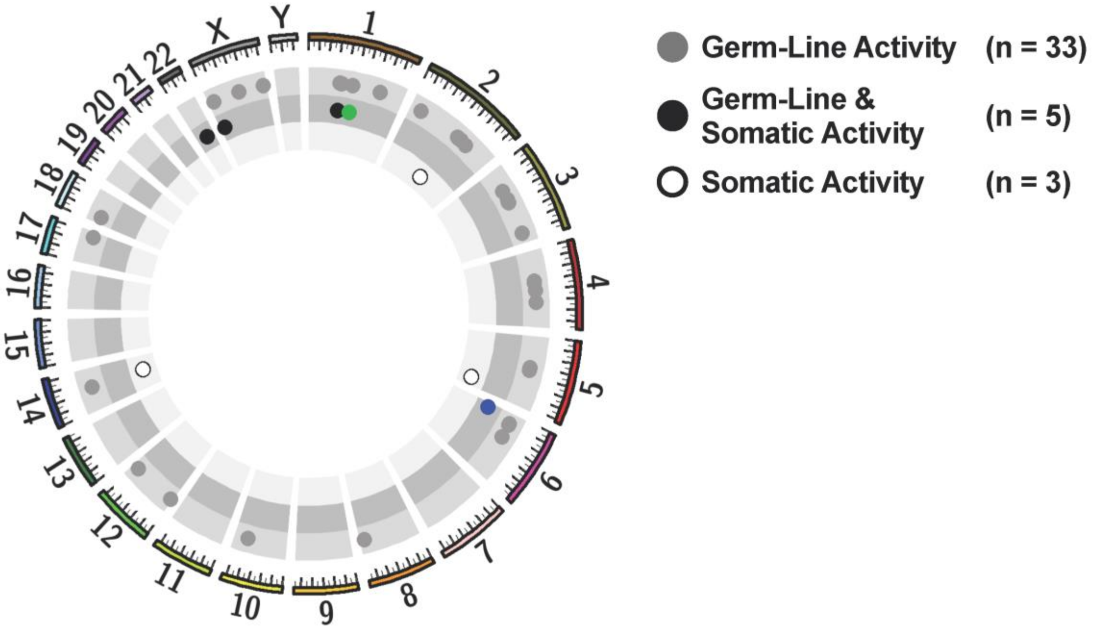

Supplemental Fig. S11. MELT sensitivity in normal/tumor pairs.

The MEIs in Figure 4 were identified with different MEI discovery tools (i.e., MELT for 1000 Genomes germline MEIs and TraFiC for somatic cancer MEIs), suggesting the possibility that variation in MEI discovery could influence these comparisons. To address this issue, we repeated these comparisons solely with MEIs that were called by MELT in both tissue types. In particular, we used MELT instead of TraFiC to rediscover somatic MEIs in four of the normal/tumor pairs that were analyzed by Tubio et al. (TCGA-AA-3516, TCGA-D5-6540, TCGA-60-2711, TCGA-66-2766; Supplemental Table S9). We then compared these somatic MEIs to the 1000 Genomes germline MEIs that we also discovered with MELT (Supplemental Table S9). MELT rediscovered 84.2% of the somatic MEIs that were identified by Tubio et al. in the cancer genomes and also rediscovered most of the differences that we observed across germline and somatic tissues. Importantly, the same three classes of L1 source elements were identified even when all of the MEIs were called with MELT (germline only, somatic only, and both). Thus, although there are slight differences in MEI detection across these platforms, L1 source elements genuinely have diverse patterns of activity in germline vs. somatic tissues.

Supplemental Fig. S12.

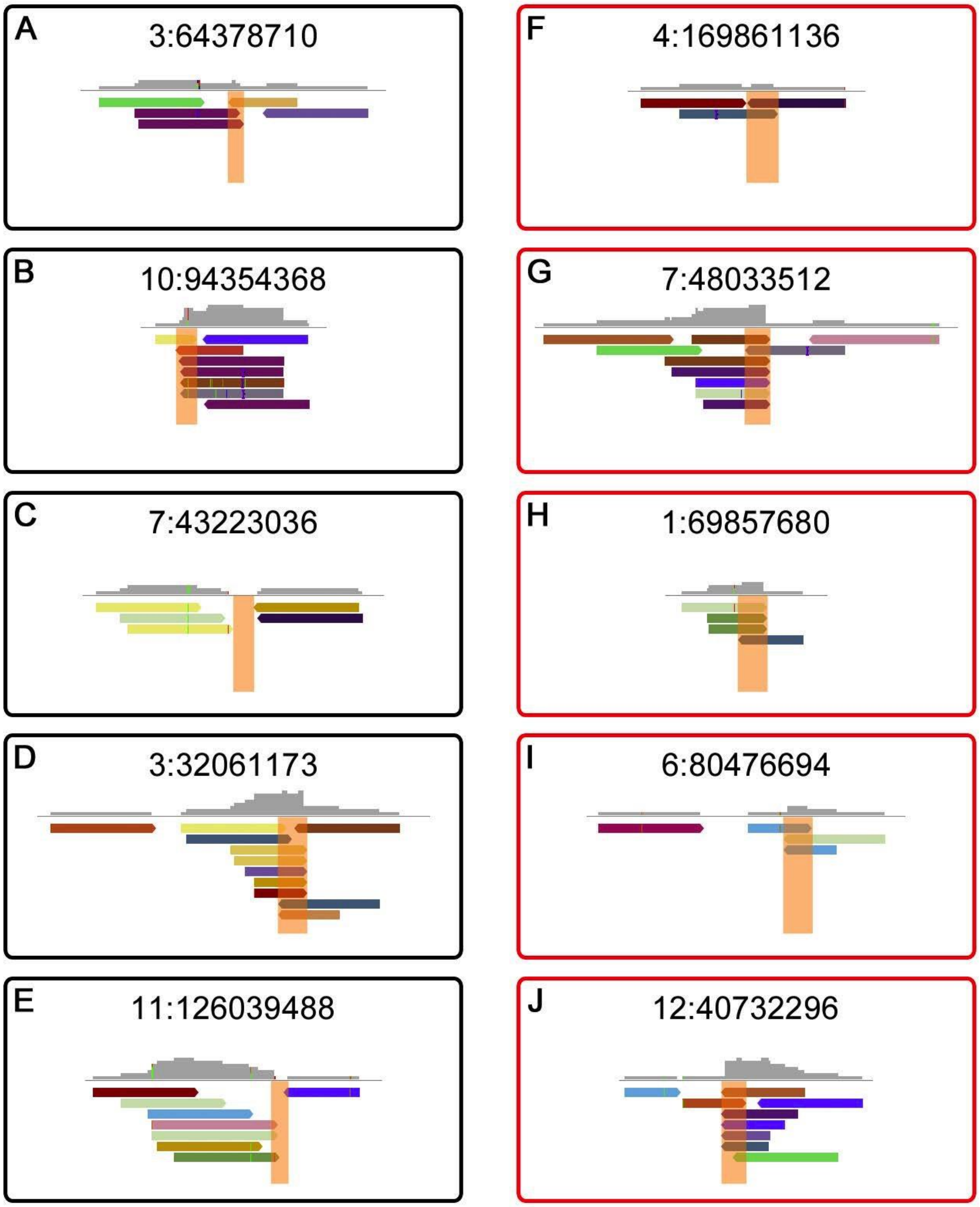

Supplemental Fig. S12. *Alu* site models for MEIs discovered in ancient genomes with MELT. Shown are IGV screen shots depicting the DRP evidence for MEI calls in ancient genomes and those called in ancient plus modern human genomes. The sites shown represent a random selection of five *Alu* sites from a much larger set of sites that were detected exclusively in ancient genomes (**A-E**; black border) or independently in ancient and modern genomes (**F-J**; red border). Note that the calls on the left (MEIs found in ancient only) and those on the right (MEIs found in both ancient and modern) have similarly strong DRP evidence supporting the calls. All data shown are for ancient MEI calls.

Supplemental Results and Discussion

Interior mutation rates in MEIs

We also leveraged the interior sequences of our *Alu* MEIs to measure the rate at which interior mutations accumulate in *Alu* copies after they are inserted into the human genome. To accomplish this goal, we examined 1,068 non-REF *Alu* MEI loci that were found in two or more of five high coverage genomes (HG00419, HG01051, HG03642, NA12878, NA19240; The 1000 Genomes Project Consortium 2015). The interior error rate that we measured across these *Alu*Y copies (2.0 X 10^{-7}) is slightly higher than the overall mutation rate that has been measured for the human genome (~1.1 X 10^{-8} ; Roach et al. 2010), but much lower than the error rate that has been measured for the L1-encoded reverse transcriptase (1.43 X 10^{-4}) (Gilbert et al. 2005). These data suggest that the error-prone L1 reverse transcriptase is the main evolutionary driver of human MEIs and their subfamilies.

Analysis of alternative poly(A) signals for 3'-transducing FL-L1 elements

The most active FL-L1 source element that produced 3' transductions in our study (*LRE3*) is a highly active "hot" L1 element that belongs to the L1-Ta1d subfamily. It has a canonical AAUAAA poly(A) signal near its native 3' end and a nearby "AC" cleavage sequence, but is lacking the downstream U-rich enhancer element (URE) (or G-rich sequence) that is often associated with functional poly(A) cleavage sites. Thus, *LRE3* preferentially uses an alternative, downstream poly(A) signal that has all of these features (AAUAAA, CA, and GUUUUG; please see Supplemental Table S9, Tab B, columns Z thru AH). The other two highly active FL-L1 source elements that produced 3' transductions in our study, the Chr1:119394974 and Chr6:13191033 elements, belong to the L1-Ta1d and L1-preTa subfamilies, respectively. Although both of these elements also had canonical AAUAAA signals near their native 3' ends, they nevertheless preferentially used alternative downstream poly(A) signals, despite the fact that neither of these sites was ideal. The downstream alternative site for the Chr1:119394974 element had a non-canonical AUUAAA poly(A) signal, lacked a CA sequence a the cleavage site, and had a URE of sequence UUUCU. The downstream alternative site for the Chr6:13191033 element had a canonical AAUAAA poly(A) signal, lacked a CA sequence, and had a URE of the sequence UUUAU. Only one of these three elements (*LRE3*) was found within a gene.

Overall, the 3'-transducing FL-L1 source elements that we identified varied considerably with respect to poly(A) signals, cleavage sites, downstream U-rich and G-rich elements, L1 subfamilies, and proximity to genes (Supplemental Table S9, Tab B, columns Z thru AH). For example, of the 38 transducing source elements in our study, 18 (47.4%) were found in the introns of known genes, and the remaining 20 (52.6%) were not located within genes. Likewise, elements from all known active L1Ta subfamilies were identified. Thus, it is unclear from these data why some elements produce greatly elevated levels of 3' transductions while others do not. A unifying theme from the literature is that the native poly(A) signals of most L1 source elements are functionally weak and are bypassed when a strong poly(A) signal is experimentally placed downstream of the original signal (Moran et al. 1996, Moran et al. 1999). Thus, it is possible that the elements in our study also have inherently weak internal signals and instead use stronger downstream signals when they are available. It is difficult to predict the relative strength of a given poly(A) signal, cleavage site, and enhancer configuration based on sequence data alone and additional experimentation will be needed to resolve this question.

FDRs in MELT ver. 1.0 vs. 2.0

As a consequence of changing the rules for MEI discovery for MELT ver. 2.0, we attained an overall increase of 36.6% in MEI detection compared to MELT ver. 1.0 (Supplemental Figs. S6A,C). We slightly altered our rules for using discordant read pairs (DRPs) and split reads (SRs), and also improved MEI detection near existing reference MEI sites. As a consequence, we detected many more rare MEIs (frequencies <0.05), and the overall frequency curves now more closely resembled the SNP frequency curve that was generated by the 1000 Genomes Project with the same

samples (Supplemental Fig. S6A). As a result of changing these rules, we lost 4 of the 11 original false positives that were detected with MELT ver. 1.0 among the 177 PCR validation sites that were examined by the 1000 Genomes Project (2 *Alu*, 1 L1, and 1 SVA; Supplemental Fig. S7E). These sites had poor trace support and were likely false positives. We also lost 13 true sites (8 *Alu*, 4 L1, 1 SVA) from the original 177 sites. We detected 7 new false positive sites (2 *Alu*, 2 L1, and 3 SVA) among the 90 new PCR validation sites that were detected with MELT ver. 2.0. Supplemental Fig. S7F displays the validation rates by allele frequency for both the old and new data. Overall, we achieved a dramatic increase in MEI detection of 36.6% while maintaining similar FDRs.

Germline vs. somatic MEIs

The 1000 Genomes Project examined germline MEIs in 2,504 genomes whereas Tubio et al. examined somatic MEIs in 244 independent cancer patients (normal/tumor pairs). Although it is possible that the status of some source elements would change upon examining a larger number of samples (germline and tumors), the concept that there will be germline only, somatic only, and shared classes is unlikely to change considerably. Some somatic source elements are themselves somatic insertions that were generated in tumors and then gave rise to new offspring. Such insertions will never be found in the germline. Also, some "germline-only" source elements are very abundant in the 1000 Genomes samples but were not found in the 244 cancer genomes that have been examined in the Tubio et al study. Thus, such elements will continue to have large differences in germline vs. somatic frequencies even if one or more somatic events is found in the future. For some tumor types where we already have abundant MEI data (e.g., lung), it is very unlikely that these trends will change radically with the addition of more samples. Therefore, although it is possible that the absolute number of germline only, somatic only and shared events will continue to evolve as new genomes are sequenced, these classes are unlikely to change significantly with the addition of more genomes.

MEIs as tools for population genetics

MEIs also have been used as genetic markers to study population genetics. Collections of ancestry informative MEI markers have been developed to track the ancestry of individuals (Sudmant et al. 2015). MEIs also have been integrated into haplotype maps by the 1000 Genomes Project, and thus, can be used to study human traits and diseases along with other forms of genetic variation. In this regard, MEIs have been identified in GWAS and eQTL studies that are linked to specific genes, suggesting that they might serve as causative variants (Sudmant et al. 2015). *De novo* MEIs, which are the youngest germline insertions in humans, have not yet been acted upon by natural selection, and thus can be more detrimental than older, more neutral MEIs.

Chimpanzee call sets.

We noted both overlap and differences in the chimpanzee MEI call sets that were generated with MELT vs. those generated by Homozdiari et al. (Supplemental Fig. S8). There are several underlying reasons for the differences in these chimpanzee MEI call sets. First, the reference genomes and alignment methods were different for the two data sets. Homozdiari et al. aligned the chimpanzee trace data to the human reference genome and used these mapping data to discover chimpanzee MEIs. In contrast, we aligned the chimpanzee trace data directly to the chimpanzee reference genome (panTro4) and used these data to detect MEIs. The Homozdiari data set also had to undergo a liftover step (from human reference genome to chimpanzee reference genome coordinates) in order to compare the data sets and some sites were altered during this step (this was done by us to compare datasets on the same reference coordinates). Finally, the MEI discovery tools were different: Homozdiari et al. used a tool that they developed (a modified version of their Variation Hunter) and we used MELT.

We note that MELT detected insertions that were not detected by Homozdiari et al. and vice versa. For example, MELT detected 3,278 *Alu* insertions that were not found by Homozdiari and Homozdiari et al. detected 1,328 that were not detected by MELT. Thus, it is likely that each of these

approaches detected novel sites as a consequence of the different methods that were used (including but not limited to the different callers). This is a common occurrence when comparing any two structural variant call sets, particularly when the methods were so different. However, 4,000 *Alu* MEIs were identified with both approaches, suggesting that most of these overlapping calls are valid. Beyond these comparisons, we have no way to show the characteristics of the three sets, since we do not have mapping data for the Homozdiari calls. The MELT sites have comparable trace support and tranche distributions as our other call sets in this study.

Supplemental References

Batzer MA, Deininger PL, Hellmann-Blumberg U, Jurka J, Labuda D, Rubin CM, Schmid CW, Zietkiewicz E, Zuckerkandl E. 1996. Standardized nomenclature for Alu repeats. *Journal of Molecular Evolution* **42**: 3-6.

Beck CR, Collier P, Macfarlane C, Malig M, Kidd JM, Eichler EE, Badge RM, Moran JV. 2010. LINE-1 retrotransposition activity in human genomes. *Cell* **141**: 1159-1170.

Bennett EA, Keller H, Mills RE, Schmidt S, Moran JV, Weichenrieder O, Devine SE. 2008. Active Alu retrotransposons in the human genome. *Genome Res* **18**: 1875-1883.

Brouha B, Meischl C, Ostertag E, de Boer M, Zhang Y, Neijens H, Roos D, Kazazian HH, Jr. 2002. Evidence consistent with human L1 retrotransposition in maternal meiosis I. *Am J Hum Genet* **71**: 327-336.

Brouha B, Schustak J, Badge RM, Lutz-Prigge S, Farley AH, Moran JV, Kazazian, HH Jr. 2003. Hot L1s account for the bulk of retrotransposition activity in the human population. *Proc Natl Acad Sci USA* **100**: 5280-5285.

Danacek P, Auton A, Abecasis G, Albers CA, Banks E, DePristo MA, Handsaker RE, Lunter G, Marth GT, Sherry ST et al. 2011. The variant call format and VCFtools. *Bioinformatics* **27**: 2156-2158.

Dombroski BA, Mathias SL, Nanthakumar E, Scott AF, Kazazian HH, Jr. 1991. Isolation of an active human transposable element. *Science* **254**: 1805-1808.

Evrony GD, Cai X, Lee E, Hills LB, Elhosary PC, Lehmann HS, Parker JJ, Atabay KD, Gilmore EC, Poduri A et al. 2012. Single-neuron sequencing analysis of L1 retrotransposition and somatic mutation in the human brain. *Cell* **151**: 483-496.

Helman E, Lawrence MS, Stewart C, Sougnez C, Getz G, Meyerson M. 2014. Somatic retrotransposition in human cancer revealed by whole-genome and whole exome sequencing. *Genome Res* **24**: 1053-1063.

Holmes SE, Dombroski BA, Krebs CM, Boehm CD, Kazazian HH, Jr. 1994. A new retrotransposable human L1 element from the LRE2 locus on chromosome 1q produces a chimaeric insertion. *Nature Genet* **7**: 143-148.

Hormozdiari F, Konkel MK, Prado-Martinez J, Chiatante G, Herraez IH, Walker JA, Nelson B, Alkan C, Sudmant PH, Huddleston J et al. 2013. Rates and patterns of great ape retrotransposition. *Proc Natl Acad Sci USA* **100**: 13457-13462.

Karolchik D, Hinrichs AS, Furey TS, Roskin KM, Sugnet CW, Haussler D, Kent WJ. 2004. The UCSC Table Browser data retrieval tool. *Nucleic Acids Res* **32**: D493-496.

Kidd JM, Graves T, Newman TL, Fulton R, Hayden HS, Malig M, Kallicki J, Kaul R, Wilson RK, Eichler EE. 2010. A human genome structural variation sequencing resource reveals insights into mutational Mechanisms. *Cell* **143**: 837-847.

Konkel MK, Walker JA, Hotard AB, Ranck MC, Fontenot CC, Storer J, Stewart C, Marth GT, Batzer MA. 2015. Sequence Analysis and Characterization of Active Human Alu Subfamilies Based on the 1000 Genomes Pilot Project. *Genome Biol Evol* **7**: 2608-2622.

Li H, Durbin R. 2009. Fast and accurate short read alignment with Burrows-Wheeler transform. *Bioinformatics* **25**: 1754-1760.

Li H, Handsaker B, Wysoker A, Fennell T, Ruan J, Homer N, Marth G, Abecasis G, Durbin R. 2009. The sequence alignment/map format and SAMtools. *Bioinformatics* **25**: 2078-2079.

Macfarlane CM, Collier P, Rahbari R, Beck CR, Wagstaff JF, Igoe S, Moran JV, Badge RM. 2013. Transduction-specific ATLAS reveals a cohort of highly active L1 retrotransposons in human populations. *Hum Mutat* **34**: 974-985.

Meyer M, Kircher M, Gansauge MT, Li H, Racimo F, Mallick S, Schraiber JG, Jay F, Prufer K, de Filippo C et al. 2012. A High-Coverage Genome Sequence from an Archaic Denisovan Individual. *Science* **338**: 222-226.

Moran JV, Holmes SE, Naas TP, DeBerardinis RJ, Boeke JD, Kazazian HH Jr. 1996. High frequency retrotransposition in cultured mammalian cells. *Cell* **87**: 917-927.

Moran JV, DeBerardinis RJ, Kazazian HH, Jr. 1999. Exon shuffling by L1 retrotransposition. *Science* **283**: 1530-1534.

Moustafa A. 2014. JAligner: Open source Java implementation of Smith-Waterman. Vol 2014.

Myers JS, Vincent BJ, Udall H, Watkins WS, Morrish TA, Kilroy GE, Swergold GD, Henke J, Henke L, Moran JV et al. 2002. A comprehensive analysis of recently integrated human Ta L1 elements. *Am J Hum Genet* **71**: 312-326.

Ondov BD, Bergman NH, Phillippy AM. 2011. Interactive metagenomics visualization in a web browser. *BMC Bioinformatics* **12**: 385.

Ostertag EM, Kazazian HH, Jr. 2001. Twin priming: a proposed mechanism for the creation of inversions in L1 retrotransposition. *Genome Res.* **11**: 2059-2065.

Pitkanen E, Cajuso T, Katainen R, Kaasinen E, Valimaki N, Palin K, Taipale J, Aaltonen LA, Kilpivaara O. 2014. Frequent L1 retrotranspositions originating from TTC28 in colorectal cancer. *Oncotarget* **5**: 853-859.

Prado-Martinez J, Sudmant PH, Kidd JM, Li H, Kelley JL, Lorente-Galdos B, Veeramah KR, Woerner AE, O'Connor TD, Santpere G et al. 2013. Great ape genetic diversity and population history. *Nature* **499**: 471-475.

Prilic A, Yates A, Bliven SE, Rose PW, Jacobson J, Troshin PV, Chapman M, Gao J, Koh CH, Foisy S, et al. 2012. BioJava: an open-source framework for bioinformatics in 2012. *Bioinformatics* **28**: 2693-2695.

Prufer K, Racimo F, Patterson N, Jay F, Sankararaman S, Sawyer S, Heinze A, Renaud G, Sudmant PH, de Filippo C et al. 2014. The complete genome sequence of a Neanderthal from the Altai Mountains. *Nature* **505**: 43-49.

Quinlan AR. 2014. BEDTools: The Swiss-Army Tool for Genome Feature Analysis. *Current Protocols in Bioinformatics* **47**: 11.12.11-11.12.34.

Roach JC, Glusman G, Smit AFA, Huff CD, Hubley R, Shannon PT, Rowen L, Pant KP, Goodman N, Bamshad M, et al. 2010. Analysis of genetic inheritance in a family quartet by whole-genome sequencing. *Science* **328**: 636-639.

Rogers AR and Huff C. 2009. Linkage disequilibrium between loci with unknown phase. *Genetics* **182**: 839-844.

Roy AM, Carroll ML, Nguyen SV, Salem AH, Oldridge M, Wilkie AO, Batzer MA, Deininger PL. 2000. Potential gene conversion and source genes for recently integrated Alu elements. *Genome Res* **10**: 1485-1495.

Sankararaman S, Mallick S, Dannemann M, Prufer K, Kelso J, Paabo S, Patterson N, Reich D. 2014. The genomic landscape of Neanderthal ancestry in present-day humans. *Nature* **507**: 354-357.

Scott EC, Garner EJ, Masood A, Chuang NT, Vertino PM, Devine SE. 2016. A hot L1 retrotransposon evades somatic repression and initiates human colorectal cancer. *Genome Res.* **26**: 745-755.

Solyom S, Ewing AD, Hancks DC, Takeshima Y, Awano H, Matsuo M, Kazazian HH, Jr. 2012a. Pathogenic orphan transduction created by a nonreference LINE-1 retrotransposon. *Human Mutation* **33**: 369-371.

Solyom S, Ewing AD, Rahrmann EP, Doucet T, Nelson HH, Burns MB, Harris RS, Sigmon DF, Casella A, Erlanger B et al. 2012b. Extensive somatic L1 retrotransposition in colorectal tumors. *Genome Res* **22**: 2328-2338.

Sudmant PH, Rausch T, Gardner EJ, Handsaker RE, Abyzov A, HuddlestonJ, Zhang Y, Ye K, Jun G, His-Yang Fritz M, et al. 2015. An integrated map of structural variation in 2,504 human genomes. *Nature* **526**: 75-81.

The 1000 Genomes Project Consortium. 2015. A global reference for human genetic variation. *Nature* **526**: 68-74.

Tubio JM, Li Y, Ju YS, Martincorena I, Cooke SL, Tojo M, Gundem G, Pipinikas CP, Zamora J, Raine K, et al. 2014. Mobile DNA in cancer: Extensive transduction of nonrepetitive DNA mediated by L1 retrotransposition in cancer genomes. *Science* **345**: 1251343.

Zhao J, Hyman L, Moore C. 1999. Formation of mRNA 3' ends in eukaryotes: mechanism, regulation, and interrelationships with other steps in mRNA synthesis. *Microbiol Mol Biol Rev* **63**: 405-445.
